# Exploring Species Level Taxonomy and Species Delimitation Methods in the Facultatively Self-Fertilizing Land Snail Genus *Rumina* (Gastropoda: Pulmonata)

**DOI:** 10.1371/journal.pone.0060736

**Published:** 2013-04-05

**Authors:** Vanya Prévot, Kurt Jordaens, Gontran Sonet, Thierry Backeljau

**Affiliations:** 1 Royal Belgian Institute of Natural Sciences, Brussels, Belgium; 2 Laboratoire d’Evolution Biologique et Ecologie, Université Libre de Bruxelles, Brussels, Belgium; 3 Evolutionary Ecology Group, University of Antwerp, Antwerp, Belgium; 4 Joint Experimental Molecular Unit (JEMU), Royal Museum for Central Africa, Tervuren, Belgium and Royal Belgian Institute of Natural Sciences, Brussels, Belgium; Biodiversity Insitute of Ontario - University of Guelph, Canada

## Abstract

Delimiting species in facultatively selfing taxa is a challenging problem of which the terrestrial pulmonate snail genus *Rumina* is a good example. These snails have a mixed breeding system and show a high degree of shell and color variation. Three nominal species (*R. decollata, R. saharica* and *R. paivae*) and two color morphs within *R. decollata* (dark and light) are currently recognized. The present study aims at evaluating to what extent these entities reflect evolutionary diverging taxonomic units, rather than fixed polymorphisms due to sustained selfing. Therefore, a phylogenetic analysis of nuclear (ITS1, ITS2) and mitochondrial DNA (COI, CytB, 12S rDNA, 16S rDNA) sequences was performed. Putative species in *Rumina,* inferred from the mitochondrial DNA phylogeny, were compared with those proposed on the basis of the COI gene by (1) DNA barcoding gap analysis, (2) Automatic Barcode Gap Discovery, (3) the species delimitation plug-in of the Geneious software, (4) the Genealogical Sorting Index, and (5) the General Mixed Yule Coalescent model. It is shown that these methods produce a variety of different species hypotheses and as such one may wonder to what extent species delimitation methods are really useful. With respect to *Rumina*, the data suggest at least seven species, one corresponding to *R. saharica* and six that are currently grouped under the name *R. decollata*. The species-level status of *R. paivae* is rejected.

## Introduction

Since the Convention on Biological Diversity (Rio “Earth Summit”, 1992) and with the recent booming of DNA barcoding [1], taxonomy has been pushed upward as the discipline that delimits the basic units with which biodiversity is commonly measured, viz. “species” [2], [3], [4]. However, there is no consensus about the meaning of this latter term [3], [5] and currently there are more than 25 different species concepts [6], [7], none of which seems to be universally applicable [5].

Usually it is not even clear under which concept taxonomists describe new species, which weakens the idea that species descriptions represent scientific hypotheses [8], [9]. Nevertheless, hitherto most metazoans have been implicitly described as “morphospecies”, while later on they almost automatically have been regarded as “biological species” [10].

The interpretative shift from “morphospecies” to “biological species” assumes that the morphological differences between species reflect reproductive isolation among outcrossing populations. However, this assumption may be severely flawed in (facultative) uniparental taxa which, through parthenogenesis or autogamy, may produce taxonomically deceiving phenotypic divergence, due to the fixation of alternative alleles in different strains or multilocus genotypes (MLGs; we will use the terms “strain” and “MLG” interchangeably) [10], [11], [12]. Such phenotypically divergent strains can easily be taken for “morphospecies”, especially if (1) occasional outcrossing between strains is rare and/or geographically, ecologically or temporarily patterned [12], [13], (2) phenotypic differences are due to dominant/recessive, linked and/or pleiotropic alleles [14], [15] and (3) allelic combinations are adaptively constrained [16], [17]. Obviously, under these circumstances morphological differences may be misleading if used to delimit biological species (see [10] for a case in point). Conversely, interpreting morphological differences among (groups of) strains as mere intraspecific polymorphisms ignores that such differentiation may reflect historical divergences that are consistent with the idea that species are evolving lineages or populations [6], [18], [19], [20], [21].

A particularly striking example of the difficulties that arise when trying to delimit species in facultatively selfing animals taxa is provided by the hermaphroditic, decollate terrestrial snails of the genus *Rumina* Risso, 1826 (family Subulinidae). Morphological differentiation in this genus has been interpreted either as reflecting different nominal species [22], [23] or as representing selfing strains, varieties or ecophenotypes within a single species [16], [17], [24], [25]. Referring to the first interpretation and based on differences in shell shape, color and size, several nominal *Rumina* species have been described, three of which are currently still recognized as “good” species, viz. *R. decollata* (Linnaeus, 1758), *R. paivae* (Lowe, 1860), and *R. saharica* Pallary, 1901 [22], [23]. These three species are indigenous in the Mediterranean area [26], with *R. decollata* occurring all over the West Mediterranean region (East Adriatic coast, Italy, South France, Iberian peninsula, North Africa), *R. paivae* only occurring in North Africa (Morocco, Algeria, Tunisia), and *R. saharica* living in the Eastern and Southeastern part of the Mediterranean region (Greece, West and South Turkey, Syria, Lebanon, Egypt and Libya) [24], [27]. *Rumina decollata* has also been introduced in many other areas in the world [28].

Because of their more or less complementary distributions and their morphological similarity, the three *Rumina* species have previously also been considered as varieties or subspecies of a single species [22], [24], [27]. Yet, since *R. decollata* and *R. saharica* maintain their morphological differences in sympatry, they have been raised to species rank [22]. Obviously, this implicitly assumes reproductive isolation under the biological species concept (BSC). For *R. paivae*, of which the original description was based on two empty shells from Rabat (Morocco) (Lowe, 1860), no rationale was provided for its re-valorization as a separate species, except for an outline of conchological differences [23], [29].

Prior to the re-valorization of *R. decollata, R. saharica* and *R. paivae* as separate nominal species, allozyme studies showed that *R. decollata* is a complex of >30 homozygous strains [17] with most natural populations consisting of single, or only very few, strains [16], [17], [25]. These observations were interpreted as if *R. decollata* was a facultative, if not predominant, self-fertilizer [25], [32], despite repeated observations of mating behavior [30], [31] and occasional records of individuals with heterozygous allozyme genotypes [16]. As such, the strains of *R. decollata*, or groups thereof, may show fixed allelic differences at up to more than half the number of allozyme loci surveyed [16], [25]. Several of these highly divergent allozyme strains also reveal consistent differences in color, shell width and egg weight, so that the strains (or groups thereof) might represent different subspecies or varieties of *R. decollata* [25]. It was even observed (1) that insofar *R. decollata* shows mating behavior, it tends to do so assortatively according to color morph and (2) that the amount of allozyme divergence between these color morphs in Southern France is of the same magnitude as an average pair of sibling *Drosophila* or rodent species [16]. Nevertheless, these issues were interpreted as mere intraspecific polymorphisms, reflecting adaptation and competition between facultatively selfing organisms [17]. Obviously, if self-fertilization in *R. decollata* can produce allozymically highly divergent strains that differ in body color, life history and microhabitat to such degree that they may mimic different species, then one could assume that also *R. paivae* and *R. saharica* may represent divergent strains produced by (facultative) selfing, rather than separate species.

Against this background, the present paper explores to what extent the nominal species *R. decollata*, *R. paivae* and *R. saharica*, and the two color morphs (“dark” and “light”) of *R. decollata* [16], reflect evolutionary divergent lineages. To this end, we first conducted a phylogenetic analysis of nuclear and mtDNA nucleotide sequences in *Rumina* to define molecular operational taxonomic units (MOTUs, sensu Blaxter, 2004 [33]) under a phylogenetic species concept (PSC or more generally under a lineage concept, in which a species is viewed as a definitively diverging lineage [34]). Then, species limits were explored and compared with five species delimitation methods applied to COI DNA barcodes. We also constructed species trees to explore eventual incongruences with the gene trees [35]. Finally, we compared the outcome of the various methods and markers in an attempt to re-interpret the taxonomy of the genus *Rumina*.

## Materials and Methods

### Specimen Collection

**Figure 1:**
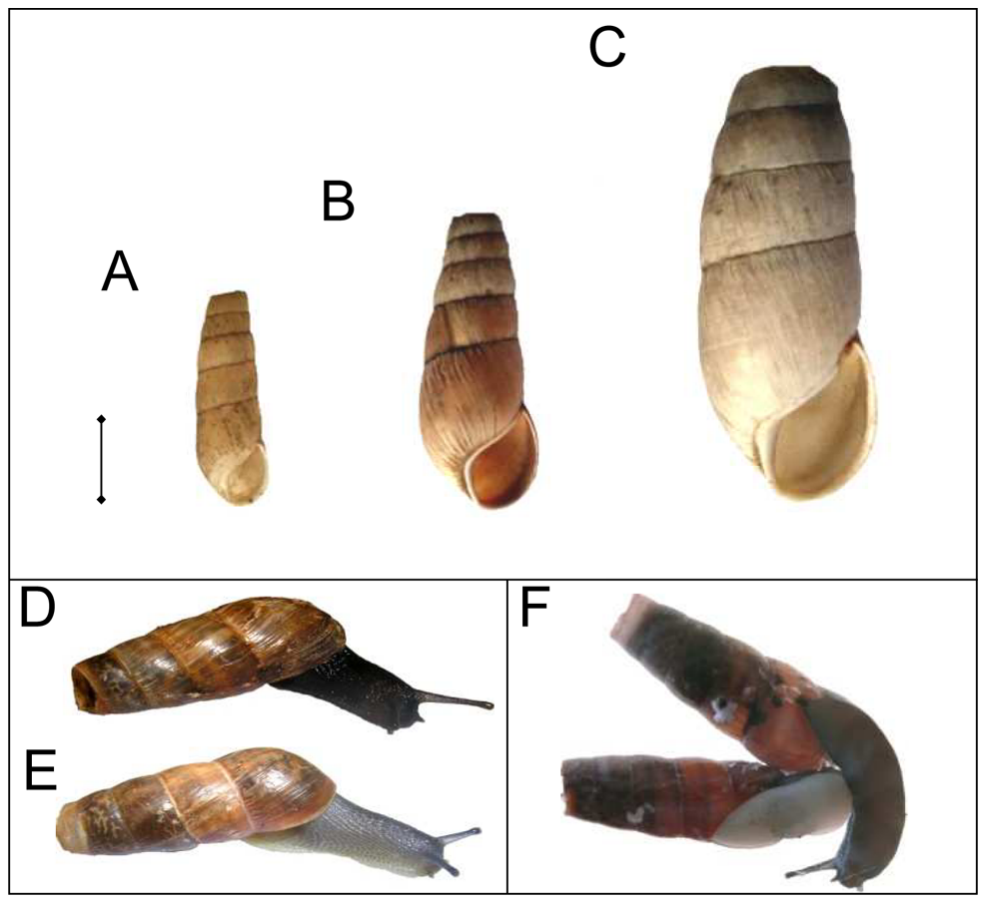
Shells of *Rumina* spp. A) *R. saharica,* B) *R. decollata,* C) *R. paivae,* D) dark phenotype of *R. decollata* (MOTU A), and E) light phenotype of *R. decollata* (MOTU Eb). Scale = 1 cm.

A total of 458 specimens of Rumina sp., involving 396 R. decollata (including the light and dark morphs [16]), four R. paivae, and 58 R. saharica (Figure 1), representing 68 populations from nearly all over the Mediterranean region, were sampled for DNA sequence analysis (Table S1). Snails were hand-picked in the field and either kept alive until they were stored at –80°C or immediately placed in 70% ethanol. Live specimens were photographed and scored for the color of the foot, body and shell. Species were identified according to [22], [29]. Rumina saharica has a slender and more cylindrical shell with a smaller aperture than both other species (Figure 1). The body and shell are cream-colored in R. saharica and varies from light grey or pale brown to black in R. decollata. The dark morph of R. decollata has a black body and a dull olive-gray foot, whereas the light morph has a light gray body with a medio-dorsal black line and a pale yellowish foot [16]. Rumina paivae (body color unknown) has a similar shell shape as R. decollata, but its shell width exceeds 15.5 mm [29]. All the material is deposited in the collections of the Royal Belgian Institute of Natural Sciences, Brussels, under catalogue number IG 31 791. No specific permits were required for the described field studies. All samples were collected and treated in accordance with legal regulations and property rules. Moreover, the genus Rumina does not involve endangered or protected taxa.

### DNA Extraction and PCR Amplification

Total DNA was extracted from a small piece of the foot using the NucleoSpin®Tissue Kit (Macherey-Nagel, Düren, Germany) according to the manufacturer’s instructions. PCR amplifications were done in 25 µl reaction volumes containing 2 µM of each dNTP (GE Healthcare, Buckinghamshire, U.K.), 0.2 µM of each primer, 1.25 U of Taq® DNA Polymerase (Qiagen, Venlo, The Netherlands), 1× PCR Buffer (Qiagen, Venlo, The Netherlands), and 1 µl of DNA extract. Four mtDNA gene fragments (12S rDNA, 16S rDNA, COI and CytB) and two complete nuclear ribosomal internal transcribed spacers (ITS1 and ITS2) were amplified with the primers listed in Table 1. PCR always started with a denaturation step of 95°C for 5 min, followed by the cycles described in Table 1, and always stopped with a final elongation step at 72°C for 5 min. All amplifications included positive and negative controls. PCR products were purified using the NucleoFast®96PCR Plate (Macherey-Nagel, Düren, Germany) following the manufacturer’s instructions. Purified DNA was recovered in 30 µl of sterilized water.

**Table 1:** Gene fragments, conditions used for PCR and sequencing and forward (F) and reverse (R) PCR primer sequences (5′-3′).

Gene	PCR cycle conditions	Primers
12S^a^	40 cycles: 45 s at 95°C, 45 s at 51°C	F: AAACTGGGATTAGATACCCCACTAT
	and 2 min at 72°C	R: GAGGGTGACGGGCGGTGTGT
16S^b^	35 cycles: 45 s at 95°C, 45 s at 40°C	F: CCGGTCTGAACTCAGATCACGT
	and 1 min at 72°C	R: CGCCTGTTTAACAAAAACAT
COI^c^	35 cycles: 45 s at 95°C, 45 s at 40°C	F: GGTCAACAAATCATAAAGATATTGG
	and 1 min at 72°C	R: TAAACTTCAGGGTGACCAAAAAATCA
CytB^d^	40 cycles: 45 s at 95°C, 45 s at 45°C	F: TGTGGRGCNACYGTWATYACTAA
	and 2 min at 72°C	R: AGGAARTAYCAYTCNGGYTG
ITS1^e^	35 cycles: 1 min at 95°C, 1 min at 55°C	F: TCCGTAGGTGAACCTGCGGAAGGAT
	and 2 min at 72°C	R: TGCGTTCAAGATATCGATGTTCAA
ITS2^f^	30 cycles: 30 s at 94°C, 30 s at 50°C	F: CATCGACATCTTGAACGCATA
	and 1 min at 72°C	R: TTCACTCGCCGTTACTAGGG

a Modified from Kocher et al., 1989 [97].

b Palumbi, 1996 [98].

c Folmer et al., 1994 [99].

d Modified from Merritt et al., 1998 [100].

e Hillis and Morris, 1991 [101].

f Wade et al., 2006 [102].

### DNA Sequencing

Cycle-sequencing was performed with the BigDye® Terminator v. 1.1 cycle Sequencing kit (Applied Biosystems, Lennik, Belgium), using the PCR primers. Sequencing was done with an ABI 3130xl capillary DNA sequencer (Applied Biosystems). Sequences were deposited in GenBank (Table S1).

### Cloning

Because some sequence readings of ITS1 and ITS2 showed overlapping peak patterns, suggesting intra-individual sequence heterogeneity, we cloned the corresponding PCR products using the TOPO® TA Cloning Kit (Invitrogen, Merelbeke, Belgium) according to the manufacturer’s instructions. Eight colonies were picked per specimen and put into 50 µl of 0.85% PBS. Inserts were checked by PCR amplification of 2.5 µl of PBS-colony in a total of 25 µl PCR mix, applying the same PCR-conditions as described above. Purification and sequencing of the PCR products was also done as described earlier.

### Phylogenetic Analysis and Species Delimitation

Both forward and reverse DNA sequences were analyzed in SeqScape v. 2.5 (Applied Biosystems) and alignments were made using the default parameters of the IUB scoring matrix of the ClustalW algorithm in BioEdit v. 7.0.8 [36]. COI and CytB protein coding sequences were translated into amino acids, using MEGA v. 4.0 [37], to check for stop codons. Phylogenetic analyses were conducted both on the separate and concatenated mitochondrial (cmtDNA = 12S +16S+COI+CytB) or nuclear (cITS = ITS1+ ITS2) data sets. Basic sequence statistics of the alignment were calculated in DnaSP v. 5.00.07 [38] (Table 2). Substitution saturation was assessed using Xia et al.’s (2003) [39] test, implemented in DAMBE v. 4.2.13 [39], [40].

**Table 2:** Sequence information for the different gene fragments without the outgroup (values with the outgroup in parentheses).

Gene fragments		Length	PS	PI	G/M	jModelTest	alpha	MrModeltest
mtDNA	12S	403	155	145	69	GTR+G	0.4253	GTR+G
		(410)	(183)	(178)	(88)			
	16S	473	122	113	84	GTR+G	0.2093	GTR+G
		(479)	(153)	(146)	(92)			
	COI	655	233	223	2	TVM+I+G	0.9476	GTR+I+G
		(655)	(254)	(244)	(4)			
	COI-position1	–	–	–	–	–	–	GTR+G
	COI-position2	–	–	–	–	–	–	F81
	COI-position3	–	–	–	–	–	–	HKY+G
	CytB	361	178	171	0	TPM1uf+I+G	1.9807	GTR+I+G
		(362)	(148)	(178)	(0)			
	CytB-position1	–	–	–	–	–	–	GTR+I+G
	CytB-position2	–	–	–	–	–	–	HKY+G
	CytB-position3	–	–	–	–	–	–	HKY+G
	**cmtDNA**	1892	688	652	155	TVM+G	0.8675	GTR+I+G
		(1906)	(738)	(746)	(184)			
ITS	ITS1	758	25	24	203	TPM2uf+G	0.4967	GTR+G
		(782)	(173)	(24)	(258)			
	ITS2	508	15	9	14	TPM3uf+G	0.3036	HKY+G
		(517)	(98)	(12)	(82)			
	**cITS**	1266	40	33	217	TPM2uf+G	0.5780	GTR+G
		(1299)	(271)	(38)	(340)			

Fragment length in bp, number of variable sites (PS), number of parsimony informative sites (PI), and number of gap positions or missing data (G/M; see text). On the right side: models selected by the Akaike Information Criterion [44] in the programs jModelTest v. 0.1.1 and MrModeltest v. 2.2 for Bayesian analysis, and alpha value for the substitution models.

Phylogenetic trees were inferred by Neighbor-Joining (NJ), Maximum Parsimony (MP), Maximum likelihood (ML) and Bayesian inference (BI). Trees were rooted with the land snail *Subulina octona* (Subulinidae). Nucleotide substitution models were selected for each gene separately, for 1^st^, 2^nd^ and 3^rd^ codon positions in COI and CytB, and for each entire concatenated dataset (ML). Concatenated datasets for BI trees were partitioned with appropriate models applied to each partition. Model selection was done with jModelTest v. 0.1.1 [41] for ML, and MrModeltest v. 2.2 [42] in conjunction with PAUP*, v. 4.0b10 [43] for BI. The best fitting models were chosen with the Akaike Information Criterion [44].

NJ trees were constructed with MEGA v. 4.0 using the Kimura 2-parameter (K2P) distance, with complete deletion of missing-information and alignment gaps (indels).

MP trees were inferred in PAUP* using a heuristic search. Alignment gaps were treated as missing data.

ML trees were constructed using PhyML v. 3.0 [45]. Four substitution rate categories were considered, while gamma shape parameters, transition/transversion ratios, and nucleotide frequencies were estimated from the data. Proportions of invariable sites were set according to the values given by the models obtained with jModelTest v. 0.1.1 (Table 2). Alignment gaps were treated as unknown characters.

BI trees were constructed with MrBayes v. 3.1.2 based on a cold chain and three incrementally heated chains with *T* = 0.2, running for 4,500,000 generations with a sample frequency of 200. The first 25% of the trees were discarded and the remaining trees were used to build a consensus tree and estimate Bayesian posterior probabilities (PP). By this time the chains had all converged to a stable standard deviation of split frequencies <0.01. Eight partitions corresponding to 12S, 16S and each codon position in COI and CytB were set and run with their best fitting substitution models. Two independent runs were executed in order to ensure that analyses were not trapped in local optima. Alignment gaps were treated as missing data.

Branch support was considered to be meaningful if ≥70% for bootstrapping (BS) [46] (based on 1000 replicates) or P≥0.95 PP [47]. Although alignment gaps were not considered when reconstructing trees, we did use them subsequently as additional qualitative data to support and diagnose MOTUs.

Putative species limits were explored with five methods, viz. (1) “classical” DNA barcoding gap analysis [1], [48], [49], (2) the Automatic Barcode Gap Discovery (ABGD) [50], (3) the species delimitation plug-in (SDP) [51], (4) the Genealogical Sorting Index (GSI) [52], and (5) the General Mixed Yule Coalescent (GMYC) model [53]. These methods were only applied to the COI sequence data because this fragment is routineously used to identify animal species (i.e. “DNA barcoding” [54]) and has been employed for developing species delimitation methods (e.g. [50]).

Details on the implementation of these five methods are provided here below.

A “classical” DNA barcoding gaps analysis was based on K2P distances [1], [54], even though the general use of K2P as a standard model for DNA barcoding gap analysis is debated [55], [56], [57]. DNA barcode gaps were assessed with the APE package v. 2.6-1 [58] and the graphics functions in Bioconductor [59]. This was done in two ways: (1) by determining barcode gaps between single well-supported MOTUs and amalgamations of all other MOTUs within nominal taxa (e.g. MOTU X vs all other MOTUs in *R. decollata*) and (2) by determining pairwise barcode gaps between single well-supported MOTUs (e.g. MOTU X vs MOTU Y). The first method will be referred to as “overall gap analysis” (OGA), the second as “pairwise gap analysis” (PGA).

The ABGD method [50] automatically finds the distance at which a barcode gap occurs and sorts the sequences into putative species based on this distance [50]. Briefly, the method statistically infers the barcode gap from the data and partitions the data accordingly. This procedure is then recursively applied to the previously obtained groups of sequences [50]. COI alignments were uploaded at http://wwwabi.snv.jussieu.fr/public/abgd/abgdweb.html and ABGD was run with the default settings (Pmin = 0.001, Pmax = 0.1, Steps = 10, X (relative gap width) = 15, Nb bins = 20) and with K2P distances.

The SDP [51] in the Geneious software (http://www.biomatters.com) (1) evaluates the phylogenetic exclusivity of each putative species interpreted as a clade by testing the probability that this exclusivity or monophyly has occurred by chance in a coalescent process, and (2) assesses the probability with which a putative species can be diagnosed successfully on a phylogenetic tree by comparing intra- and interspecific genetic distances [51]. SDP was used to calculate *Rosenberg’s P_AB_* [60], a test for taxonomic distinctiveness based on the null hypothesis that monophyly is a chance outcome of random branching, and *Rodrigo’s P(Randomly Distinct)* [*Rodrigo’s P(RD)*] [61], which is the probability that a clade has the observed degree of distinctiveness (i.e. the ratio between the distance from a species-defining node to the tips of the tree, and the distance from that same node to its immediate ancestor [51]) due to random coalescence.

The GSI [52] explores species limits by measuring the degree of exclusive ancestry of a group on a rooted tree topology, where a group corresponds to a set of branch tips with the same label and exclusivity is the “amount” of ancestry for a group that is common to only members of the group [52]. As such, GSI measures exclusive ancestry on a scale (index, where a monophyletic group has a value of 1) and the level of support (p-value) for this score [52]. Both BI and ML trees for the COI data were uploaded into the online program at http://www.genealogicalsorting.org. Groups were labeled according to the MOTUs retrieved from the phylogeny (see results), and the GSI was calculated using 10,000 permutations [52]. Statistical tests were corrected for multiple test biases using the sequential Bonferroni procedure [62].

The GMYC model [53] estimates the transition from coalescent to speciation branching patterns on a Bayesian ultrametric tree. In brief, the method identifies the most likely point (threshold) where there is a transition of branching rates (within and between species) and compares the likelihood of the GMYC model with a null model assuming that all sequences are derived from a single species [53], [63]. We used the COI BI tree that was transformed into an ultrametric, fully dichotomous tree (polytomies were resolved using zero-length branches) using Mesquite v. 1.12 [64] (convergence was not achieved under a relaxed lognormal clock inference in BEAST v. 1.6.1 [65]). Putative species were identified using the single- and multiple-threshold GMYC model as implemented by the SPLITS program in R (Species’ LImits by Threshold Statistics, version 2.10, https://r-forge.r-project.org/projects/splits/).

We used the Species Tree Ancestral Reconstruction (∗BEAST) [66] method implemented in BEAST v. 1.6.1 [65] to look for congruence between the BI tree topologies of the cmtDNA and cITS data.*BEAST operates under a Bayesian framework, co-estimating the posterior distribution of species and gene trees using a coalescent model. The method combines priors for speciation events and population genetic, allowing to account for intraspecific polymorphism and incomplete lineage sorting in the phylogenetic estimation procedures [66]. The *BEAST analysis ran for 100,000,000 generations with a sample frequency of 10,000. We used a lognormal clock (without fossil calibrations) and a mean rate fixed to one. The Yule tree prior was used for species-level analyses and a constant coalescent model was used for population-level analyses. Models of DNA sequence evolution were assigned to each partition based on results from MrModeltest (same substitution models used in BI analysis). Default values were used for remaining priors. The final ∗BEAST species tree was a maximum clade credibility tree with median node heights after burnin of 10,000 trees. Convergence was confirmed in Tracer v. 1.5 [67], with the species tree reconstructed using Tree Annotator v. 1.6.1 [65]. Support for nodes was determined using PP.

Finally, for comparison with literature data, we used MEGA to calculate mean p-distances and their standard errors for cmtDNA, COI, 16S, ITS1, and the cITS data. These data are only partly shown, but the full data set is available upon request.

## Results

### Definition of MOTUs Under the PSC

#### mtDNA

The basic statistics of the mtDNA sequences and the selected substitution models are shown in Table 2. There were no stop codons in COI and CytB. None of codon positions showed signs of saturation (p = 0.00).

**Figure 2:**
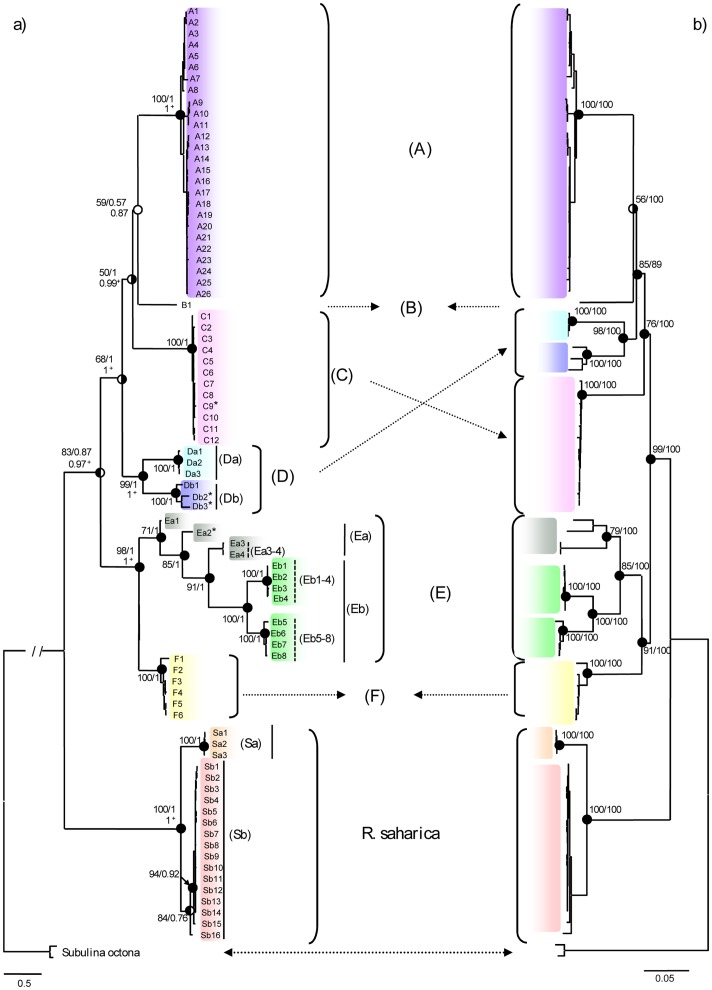
Phylogenetic trees of *Rumina* based on concatenated mtDNA sequences (12S, 16S, COI, CytB). A) BI tree with ML bootstrap values (left), BI posterior probabilities (right) and posterior probabilities of the species tree (under the line). B) NJ tree with NJ bootstrap values (left) and MP bootstrap values (right). Meaningful support (≥70% for bootstrapping [48] or p≥0.95 for posterior probabilities [49]); circles with left half black: meaningful support only for ML or NJ; circles with right half black: meaningful support only for BI or MP; white circles: no support; +: meaningful support for the species tree; *: *R. paivae* specimens. Haplotypes are listed in Table S1.

Trees based on individual gene fragments (not shown) and on the cmtDNA (Figure 2) yielded similar topologies in which (1) *R. saharica* and *R. decollata* appeared as sister taxa, (2) *R. paivae* was not monophyletic, and (3) *R. decollata* was further subdivided in five clades (A, C-F) and a single, long branch (MOTU B). Table 3 shows that MOTUs A, E and F are supported in all analyses; idem for MOTU C, except with ML of 12S. In contrast, MOTU D was only supported in 12 out of 20 cases. Moreover with BI of COI and NJ of CytB MOTUs Da and Db did not even appear as sister. All haplotypes were always assigned to the same MOTUs, except: haplotypes Ea1 from 12S and Ea1 and Ea2 from COI were placed in MOTU E in the cmtDNA trees, whereas in the single gene trees they appeared in MOTU F (we will call this MOTU F+Ea1-2).

**Table 3:** Bootstrap support for NJ, MP and ML, and posterior probabilities for BI of the different MOTUs obtained with individual gene fragments and concatenated data (cmtDNA and cITS).

	MOTU	A	B	C	D	E	F	*saharica*
cmtDNA	NJ	100	–	100	98	85	100	100
	MP	100	–	100	100	100	100	100
	ML	100	–	100	99	98	100	100
	BI	1	–	1	1	1	1	1
12S	NJ	100	–	97	77	100	100	100
	MP	100	–	100	100	100	100	100
	ML	100	–	ns	91	98	100	100
	BI	1	–	0.99	0.99	1	1	1
16S	NJ	100	–	100	73	99	100	100
	MP	100	–	100	100	100	100	100
	ML	100	–	100	ns	97	100	100
	BI	1	–	1	ns	0.96	1	1
COI	NJ	100	–	100	ns	99	100	100
	MP	100	–	100	100	100	100	100
	ML	89	–	100	ns	100	99	100
	BI	0.95	–	1	*para*	1	1	1
CytB	NJ	100	–	100	*poly*	99	100	100
	MP	100	–	100	100	100	100	100
	ML	99	–	99	ns	100	100	100
	BI	1	–	1	ns	1	1	1
cITS	NJ	93	93	94 (C+D)		79	74	98
	MP	100	75	100 (C+D)		100	100	100
	ML	100	97	93 (C+D)		77	ns	92
	BI	0.99	0.98	0.99 (C+D)		0.98	0.95	0.89
ITS1	NJ	96	ns	92 (C+D)		ns	ns	94
	MP	100	ns	100 (C+D)		100	92	100
	ML	98	ns	92 (C+D)		ns	*poly*	91
	BI	0.95	*poly*	0.97 (C+D)		0.97	*poly*	ns
ITS2	NJ	*para*	ns	ns (C+D)		ns	76	87
	MP	*para*	100	*poly*	*poly*	100	100	100
	ML	*para*	ns	ns (C+D)		ns	78	100
	BI	ns	87	87 (C+D)		ns	86	ns

ns - not-supported (i.e. if PP<0.95 or BS<70).

*poly* - polyphyletic group.

*para* - paraphyletic group.

Because of the overall similarity between the mtDNA trees, we will only focus on the cmtDNA data (Figure 2). The topology of the BI and ML cmtDNA trees (Figure 2a) differed from that of the NJ and MP trees (Figure 2b) in only four aspects: (1) The monophyly of *R. decollata* was only moderately supported in the ML tree (BS = 83) and even non-significant in the BI tree (PP = 0.87), but it was strongly supported in the NJ and MP trees (BS resp. 99 and 100); (2) The relative positions of MOTUs C and D with respect to clade (A,B) varied between the ML/BI and the NJ/MP trees; (3) The position of B as sister taxon of MOTU A was strongly supported in the MP tree (BS = 100), but not in the NJ, ML and BI trees; (4) The NJ and MP trees divided MOTU E in two well-supported sister clades, one exclusively comprising the light morph of *R. decollata* from France and Spain (MOTU Eb, i.e. the light morph [16]), the other comprising four Algerian and Tunisian haplotypes of *R. decollata* (MOTU Ea). The ML and BI trees also provided strong support for MOTU Eb, but showed MOTU Ea as a paraphyletic assemblage (Figure 2a).

Conversely, all trees showed consistently that (1) the four *R. paivae* haplotypes were distributed over MOTUs C, D and E (Figure 2). (2) MOTU A was considered to represent the dark morph [16] ] since it comprises all and only dark specimens. Note that although MOTU A almost exclusively involved specimens from France and the Iberian peninsula, it did also include the unique Maltese haplotype A23 and the Tunisian haplotype A24. (3) Except for this latter haplotype, the North African haplotypes were distributed over i) two well-supported, exclusively North African MOTUs (with MOTU C from Tunisia and one Algerian haplotype, and MOTU D from East Morocco), ii) MOTU B from West Morocco, and iii) the MOTU or paraphyletic assemblage of Tunisian and Algerian haplotypes within MOTU E. There was also a Tunisian haplotype (F2) that grouped within MOTU F. Unfortunately, since these North African specimens were not seen alive, their color could not be scored. (4) MOTU F exclusively comprised Italian and Croatian haplotypes (and haplotype F2 from Tunisia) with a dark body (like MOTU A) and a light foot (like MOTU Eb).

The numbers of different haplotypes per MOTU are shown in Table 4. In total, there were 82 cmtDNA haplotypes of which 19 belong to *R. saharica* (10 populations), four to *R. paivae* and 59 to *R. decollata* (58 populations) (Table 4). In 27 *Rumina* populations (ca. 40%) we observed more than one haplotype, with a maximum of five co-occurring haplotypes in population Grc1 (*R. saharica*). Some populations comprised haplotypes from different MOTUs: haplotypes of MOTUs A and Eb co-occurred in populations FM3, FmtB1, FmtC1 and FmtB2, haplotypes of MOTUs C and Ea, co-occurred in population Tun1, and haplotypes of clades Ea and F co-occurred in population Tun5.

**Table 4:** Mean p-distances and their standard errors between and within (diagonal) the different MOTUs based on the cmtDNA data.

	*R. decollata*							*R. saharica*
	MOTU	A	B	C	D	E	F	
	**A (26)**	0.012±0.001						
	**B (1)**	0.119±0.007	n/c					
***R. decollata***	**C (12)**	0.143±0.008	0.122±0.008	0.004±0.001				
**(63)**	**D (6)**	0.136±0.007	0.130±0.007	0.141±0.007	0.076±0.005			
	**E (12)**	0.164±0.008	0.164±0.008	0.163±0.007	0.165±0.007	0.078±0.004		
	**F (6)**	0.151±0.008	0.146±0.008	0.146±0.008	0.159±0.007	0.148±0.007	0.010±0.001	
***R. saharica*** ** (19)**		0.198±0.009	0.186±0.009	0.189±0.009	0.196±0.009	0.201±0.009	0.196±0.009	0.020±0.002

Note that the four *R. paivae* haplotypes are included in *R. decollata*. Numbers of haplotypes are indicated in parentheses.

n/c - not computable.

The species tree of the mtDNA data (not shown) was congruent with the BI gene tree of the cmtDNA data set (Figure 2a) and all the MOTUs defined by the cmtDNA were supported.

Plotting indels on the trees provided extra support for several MOTUs as follows (Table 5A): the MOTUs of *R. saharica* were consistently differentiated from *R. decollata* (overall) at 21 positions involving indels, while MOTUs Sa and Sb within *R. saharica* were consistently differentiated at six indel positions in 12S and 16S. Within *R. decollata*, MOTUs A, B, C, Da, E, Ea, Eb and F had characteristic indels in 12S and/or 16S. The *R. paivae* individuals didn’t show specific indels.

**Table 5:** Positions of indels that are specific to the MOTUs in the cmtDNA (A) and cITS (B) data.

A)																					
12S																					
	42	43	48	126	142	147	148	169	190	198	199	200	201	250	275	276	332	352	358	359	377
A	–	–	C	–	–	A	A	A	–	–	–	–	–	–	–	–	–	T	G	–	–
B	–	–	C	G	–	–	–	A	–	A	C	C	C	–	–	–	–	A	T	–	–
C	–	–	C	A	–	–	–	A	–	–	–	–	–	T	–	–	–	C	T	–	–
D1	–	–	C	A	–	–	–	A	–	–	–	–	–	–	–	–	–	–	G	–	–
D2	–	–	T	A	–	–	–	A	–	–	–	–	–	–	–	–	–	A/G	A/G	–	–
Ea	–	–	C/T	A/G	–	–	–	A	–	–	–	–	–	–	–	–	–	T	–	–	T
Eb	–	–	T	G	–	–	–	A	–	–	–	–	–	–	–	–	–	T	–	–	–
F	–	–	C	A	–	–	–	A	–	–	–	–	–	–	–	–	–	A	G	–	–
S1	T	T	–	A	T	–	–	–	C	–	–	–	–	–	A	T	G	A	A	T	–
S2	A/T	T	T	G	T	–	–	–	C	–	–	–	–	–	–	T	G	A	A	T	–
**16S**																					
	85	170	171	172	173	206	209	219	220	222	224	316	423	424	425	453	454	455	462		
A	C	T	C	T	G	–	A	–	–	G	T	T	A	–	A/G	–	–	–	–		
B	T	A	T	T	T	–	A	A	A	G	C	T	A	–	A	–	–	–	–		
C	C	T	T	T	T	–	A	–	–	A/G	T	T	A	–	A	–	–	–	–		
D1	T	C	T	T	A	–	C	–	–	C	T	T	A	–	C	–	–	–	–		
D2	C	T	C	T	A	–	T	–	–	A/G	T	T	A	–	A	–	–	–	–		
Ea	T	C	C	A/G	T	–	C/T	–	–	A	T	T	–	–	A/G	–	–	–	–		
Eb	–	C	T	A	T	–	C	–	–	A	T	T	A	–	A/G	–	–	–	–		
F	T	T	T	G	A	–	A	–	–	A	T	T	A	T	A	–	–	–	–		
S1	G	–	–	–	–	T	–	–	–	–	G	–	T	–	–	A	A	T	–		
S2	G	–	–	–	–	T	T	–	–	–	–	–	T	–	–	A/T	A	A/T	A		
**B)**																					
	**ITS1**																	**ITS2**			
	33	63	64	65	414	440	441	442	443	444	445	460	461	556	557	558	559	102	416	417	
A	G	G	C	C	–	–	–	–	–	–	–	–	–	–	–	–	–	–	–	–	
B	G	G	C	C	–	–	–	–	–	–	–	–	–	–	–	–	–	–	–	–	
C	G	G	C	C	G	–	–	–	–	–	–	–	–	–	–	–	–	–	–	–	
D	G	G	C	C	–	–	–	–	–	–	–	–	–	–	–	–	–	–	–	–	
Ea	G	G	C	C	–	–	–	–	–	–	–	–	–	–	–	–	–	–	–	–	
Eb	G	G	C	C	–	–	–	–	–	–	–	–	–	G	C	C	C	–	–	–	
F	G	G	C	C	–	–	–	–	–	–	–	–	–	–	–	–	–	–	–	–	
S	–	–	–	–	–	G	C	A	C	C	C	G	T	–	–	–	–	G	T	T	

The separation of *R. saharica* from *R. decollata* was well supported (100% BS; PP 1.00) with a mean overall interspecific p-distance of 0.196±0.008 for cmtDNA. Intraspecific cmtDNA differentiation in *R. saharica* was much weaker than in *R. decollata* (mean p-distance of 0.020±0.002 in *R. saharica* vs. mean p-distance of 0.119±0.004 in *R. decollata*). Table 3 shows the p-distances between the different MOTUs for cmtDNA. Values ranged from 0.119±0.007 to 0.165±0.007 between the MOTUs of *R. decollata*, and from 0.186±0.009 to 0.201±0.009 between *R. saharica* and the MOTUs in *R. decollata*.

#### ITS1 and ITS2

The basic statistics of the ITS sequences and the most suitable substitution models are shown in Table 2. None of the ITS sequences showed signs of saturation (p = 0.00).

**Figure 3:**
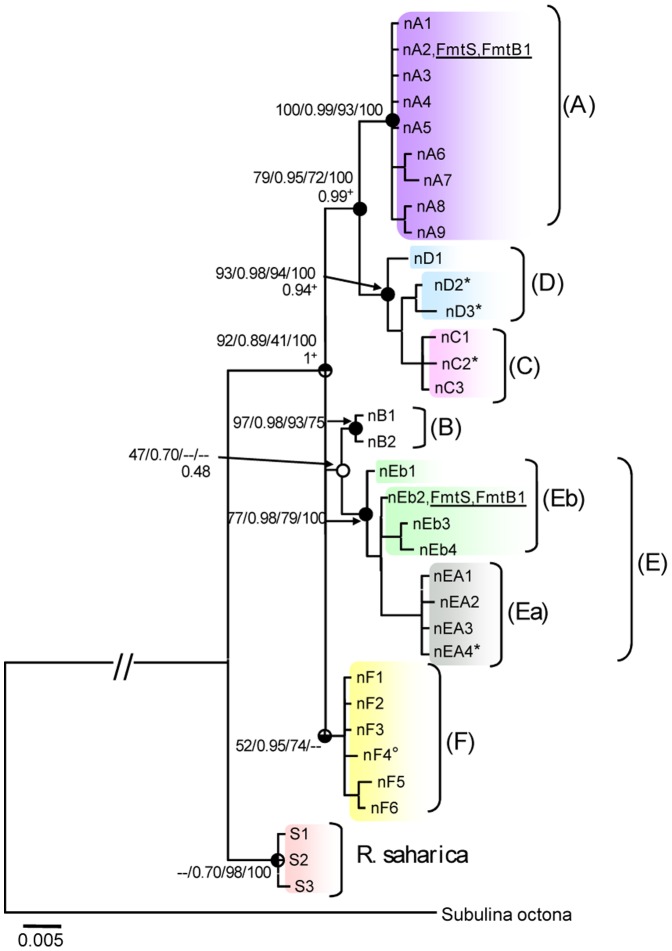
Phylogenetic trees of *Rumina* based on concatenated ITS1 and ITS2 sequences. ML bootstrap/BI posterior probabilities/NJ bootstrap/MP bootstrap values are indicated near the nodes. Posterior probabilities of the species tree are shown under the line. Meaningful support (≥70% for bootstrapping [48] or p≥0.95 for posterior probabilities [49]) is shown on dark within the circle (upper right pie: ML; lower right pie: BI; lower left pie NJ; upper left pie: MP); white circles: no support; +: meaningful support for the species tree; *: *R. paivae* specimens; °: haplotypes that are placed in other groups than with mtDNA; underlined: copies from heterozygous specimens are.

Because the tree topologies of ITS1 and ITS2 were similar, we only focused on the cITS trees (Figure 3).

The NJ, MP, ML and BI analyses of cITS yielded almost identical tree topologies (Figure 3) with the same well-supported MOTUs A, C, D, E and F as the cmtDNA tree. In addition, branch B of the cmtDNA tree was now resolved as a strongly supported MOTU of two ITS haplotypes. BS and PP values of the different MOTUs in the ITS1, ITS2 and cITS trees are shown in Table 3. Yet, there were three notable discrepancies between the cITS and cmtDNA trees: (1) cITS placed cmtDNA haplotype Ea1 in the MOTU F (nF4), while in the cmtDNA tree it turned up in MOTU Ea, (2) cITS data joined MOTUs C and D in a single, well-supported clade, and (3) MOTU Eb was not supported by cITS (but not contradicted neither). Two individuals contained two haplotypes and were therefore interpreted as putative heterozygotes: one from MOTU A and one from MOTU Eb (FmtS, FmtB1 in Figure 3).

Several nodes and MOTUs in the cITS-tree were also characterized by indels (Table 5): *R. saharica* and *R. decollata* differed by 15 indel positions, whereas MOTUs A, B, D, Ea and F shared identical indel patterns. MOTUs C and Eb where characterized by the same indel pattern but showed extra indels at position 414 (C) and at positions 556–559 (Eb).

In total there were 34 cITS haplotypes: three in R. saharica, two in R. paivae, 27 in R. decollata and two that were shared by R. paivae and R. decollata. We could not obtain ITS sequences from six populations (Alg1, PL3, PLo1, PL4, SHe and STo) and from the individuals with mtDNA haplotype F2 (Table S1). Of the 64 remaining populations, 17 (±27%) showed 2–3 cITS haplotypes, which sometimes belonged to different MOTUs.

The species tree of the ITS data (not shown) was congruent with the gene tree of the cITS data set (Figure 3) and all the MOTUs defined by the cITS were supported.

All in all, the separation between *R. saharica* and *R. decollata* was clear and well supported (ML BS = 92%; BI PP = 0.98) with a p-distance of 0.018±0.003 for cITS, of 0.023±0.005 for ITS1 and of 0.013± 0.005 for ITS2.

### Species Delimitation with COI

DNA barcoding gap analysis, SDP and GSI require pre-defined groups for testing. As such, two sets of species hypotheses were explored (Table 6): (1) those retrieved from the cmtDNA phylogeny, viz. MOTUs A, C, D, E, Ea, Eb, F and S, and (2) those retrieved from the COI data, viz. MOTUs A, C, Da, Db, Ea3-4 ( = haplotypes Ea3+ Ea4), Eb1-4, Eb5-8 and F+Ea1-2 ( = MOTU F+Ea1+Ea2) (see Figure 2 and the COI tree in the Figure S1 and Figure S2).

**Table 6:** Number of putative species delimited by the different species delimitation methods applied to the COI dataset: overall barcoding gap analysis (OGA), pairwise gap analysis (PGA), 3% threshold, stylommatophoran 4% threshold, 10× rule, 3.2–4.1× rule, ABGD method, SDP (*Rosenberg’s P_AB_* and *Rodrigo’s P(RD)*), GSI with both BI and ML trees and GMYC method.

	Classical barcoding gap analysis						ABGD	SDP				GSI		GMYC
Putative species								*P_AB_*		*P(RD)*				
	OGA	PGA	3%	4%	10×	3.2–4.1×		BI	ML	BI	ML	BI	ML	BI
**A**	+	+	+	+	−	+	+	+	−	+	−	+	+	+
**B**	na	na	na	na	na	na	na	na	na	na	na	na	na	na
**C**	+	+	+	+	+	+	+	+	+	+	+	+	+	+
**D**	−	− (B)	−	−	−	−		na	+	na	−	−	+	−
**Da** *	+	+	+	+	+	+	+	−	−	+	+	+	+	+
**Db** *	+	+	−	+	−	+	+	−	−	−	−	+	+	+
**E**	−	− (F)	−	−	−	−		+	+	−	−	−	−	
**Ea**	−	− (F)	−	−	−	−	−					−	−	−
**Eb**	+	+	−	−	−	−	+	−	−	+	−	+	+	−
**Ea3-4** *	+	+	+	+	+	+	+	−	−	+	+	+	+	+
**Eb1-4** *	+	+	+	+	+	+	−	−	−	+	+	+	+	+
**Eb5-8** *	+	+	+	+	−	+	−	−	−	+	+	+	+	+
**F**	−	− (Ea)	−	+	−	−	−					−	−	−
**F+Ea1-2** *	+	+	+	+	+	+	+	+	+	+	+	+	+	+
***R. saharica*** ** (S)**	+	+	−	+	−	+		+	+	−	−	+	+	−
**Sa**	+	+	+	+	+	+	+	−	−	+	+	+	+	+
**Sb**	+	+	+	+	+	+	+	−	−	+	+	+	+	+
**Species within ** ***R. decollata***	7 or 8	7 or 8	7	8	5	8	7	4	4	6 or 7	6	7 or 8	6 or 8	8
**Species within ** ***R. saharica***	1 or 2	1 or 2	2	1 or 2	2	1 or 2	2	1	1	2	2	1 or 2	1 or 2	2
**Species within ** ***Rumina***	8 or 10	9 or 10	9	9 or 10	7	9 or 10	9	5	5	8 or 9	8	8 or 10	7 or 10	10

+: MOTU supported as a putative species;

−: no support as a putative species; empty cases: group is not suggested or cannot be analyzed by the method; parentheses: MOTU responsible for the absence of barcoding gap;

* : additional MOTU defined by the COI data.

#### DNA barcoding gap analysis

**Figure 4:**
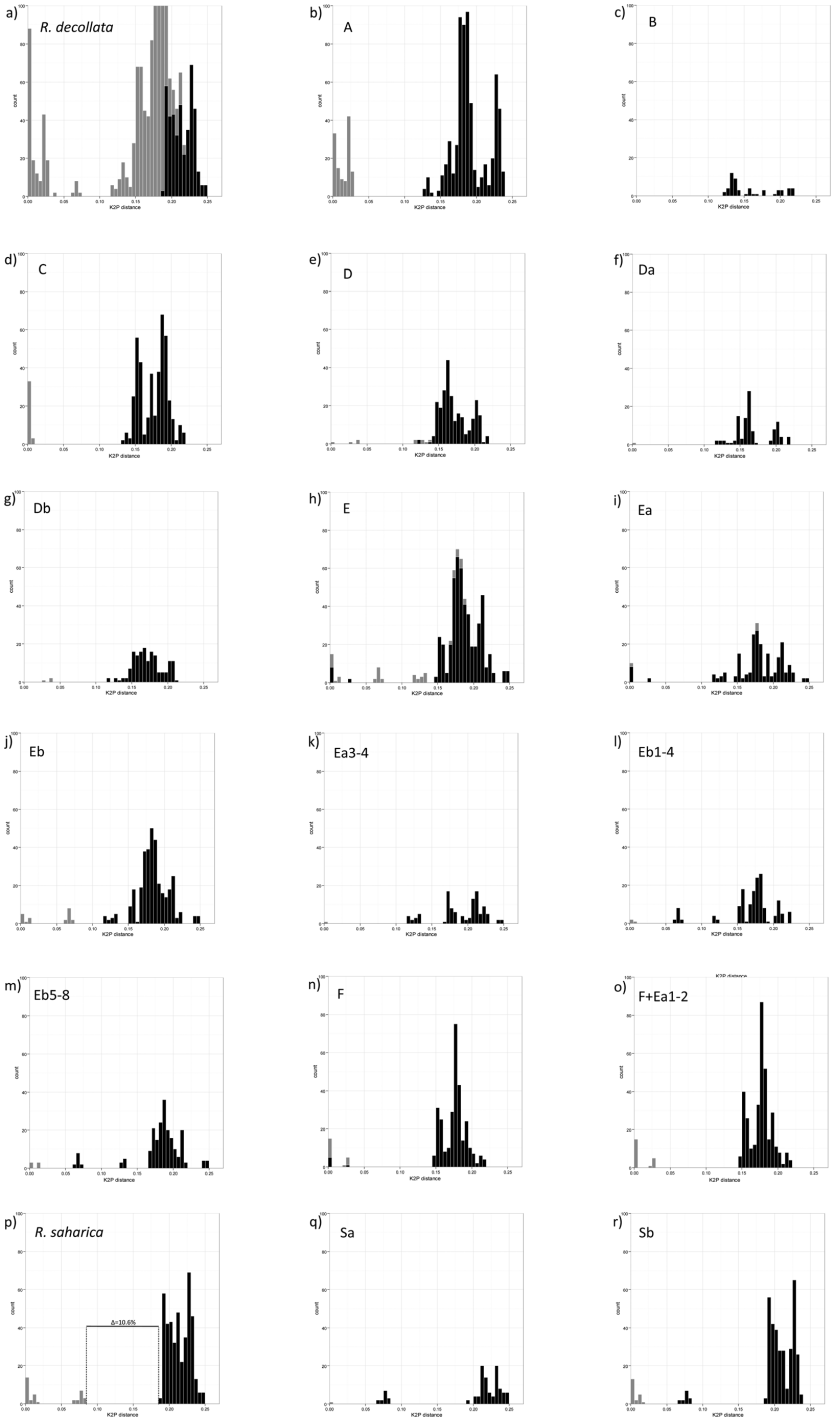
Frequency distributions of pairwise K2P distances for COI. Grey: intragroup divergences; Black: divergences between the focal group and all other groups together. Δ corresponds to the gap range. a) *R. decollata* vs *R. saharica,* b) to o) respectively MOTU A, B, C, D, Da, Db, E, Ea, Eb, Ea3-4, Eb1-4, Eb5-8, F, F+Ea1-2, vs the remaining MOTUs, p) *R. saharica* vs *R. decollata*, q) MOTU Sa vs the remaining MOTUs and r) MOTU Sb vs the remaining MOTUs.

OGA of *R. decollata* vs *R. saharica* (Figure 4a) showed considerable overlap between intra- and interspecific distances. Conversely, OGA of *R. saharica* vs *R. decollata* (Fig. 4p) showed a distance distribution over three well-separated ranges around three well-separated mean values, viz. 0.005 (0.000–0.020), 0.074 (0.065–0.083) and 0.214 (0.189–0.248). The first two values reflecting intraspecific geographic differentiation in *R. saharica*. The third value refers to the separation between *R. saharica* and *R. decollata*, corresponding to a gap range ( = interval between the highest intraspecific and the lowest interspecific distances [68], [69]) of 10.6% (Figure 4p). The mean divergence within *R. saharica* (3.3%) was considerably lower than the divergence within *R. decollata* (14.0%) or that between *R. saharica* and *R. decollata* (21.0%). Within *Rumina*, OGA showed a clear barcoding gap for MOTUs A, C, Da, Db, Eb, Ea3-4, Eb1-4, Eb5-8, F+Ea1-2, S, Sa and Sb, hence suggesting eight to ten putative species (both Eb and S were supported as one species or as two species: Eb1-4, Eb5-8 and Sa, Sb). The same groups were supported by PGA, which further allows identifying the MOTUs responsible for the absence of certain barcoding gaps: B for D, F for Ea, and Ea for F (Table 6).

#### Automatic barcode gap discovery

The ABGD method was run with a prior maximum divergence of intraspecific diversity, i.e. species divergence from 0.001 to 0.1. The number of groups for the recursive partition was nine with a prior of 0.1; 10 with 0.060, 0.036, 0.022 and 0.013; 11 for 0.008 and 0.005; 15 with 0.003 and 0.002; and 35 with 0.001. The primary partition was stable on the range of prior values with nine groups corresponding to the same groups obtained with the prior of 0.1: A, C, Da, Db, Eb, Ea3-4, F+Ea1-2, Sa and Sb (Table 6).

#### Species delimitation plugin

The results of the SDP for both BI and ML are summarized in Tables 7 and 8. Since MOTU D is paraphyletic with BI it could not be considered for the BI species delimitation. MOTU Ea also could not be tested by this method, since it doesn’t form a monophyletic group in the BI and ML trees. For both BI and ML MOTU E showed the highest *Intra/Inter* ratios (“*Intra”* is the differentiation among members of a clade, and “*Inter”* is the genetic differentiation between the members of a clade and its closest neighbor clade), indicating that the divergence within E was high relative to the divergence with the closest MOTU (Table 7). Values of *P ID(Strict)*, i.e. the mean probability that an unknown specimen of a given clade will effectively be placed within this clade (not as sister taxon to this clade) and hence will be correctly identified, were in both BI and ML higher for MOTUs A, C, F+Ea1-2, S and Sb, than for the remaining MOTUs (Table 7). *Rosenberg’s P_AB_* [60] values are significant (P<10^−5^) for MOTUs A, C, E, F+Ea1-2 and S in the BI tree and for C, D, E, F+Ea1-2 and S in the ML tree. *Rodrigo’s P(RD)* [61] values are significant (P<0.05) for MOTUs A, C, Da, Eb, Ea3-4, Eb1-4, Eb5-8, F+Ea1-2, Sa and Sb in the BI tree and for C, Da, Ea3-4, Eb1-4, Eb5-8, F+Ea1-2, Sa and Sb in the ML tree. All in all, SDP suggested five species with the *Rosenberg’s P_AB_* statistics for both BI and ML trees, but eight to nine species with the *Rodrigo’s P(RD)* for BI since both the possibilities of having one or two species (Eb1-4 and Eb5-8) in MOTU Eb were supported. Finally eight species were suggested with *Rodrigo’s P(RD)* for ML (Table 6 and 8).

**Table 7:** Summary statistics reported by the Species Delimitation plugin for COI in each putative species A) BI tree (Clade support is PP) and B) ML (Clade support is BS).

A)								
MOTU	Closest Species	*Clade Support*	*Intra*	*Inter*	*Intra/Inter*	*P ID(Strict)*	*Rosenberg's P_AB_*	*Rodrigo’s P(RD)*
A	B	0.95	0.128	0.175	0.11	0.95 (0.90, 1.0)	7.90E−17*	<0.05*
C	B	1	0.052	1.424	0.04	0.97 (0.97, 1.0)	9.60E−15*	<0.05*
D	Paraphyletic							
Da	Db	0.99	0.04	1.342	0.03	0.77 (0.60, 0.95)	6.10E−05	<0.05*
Db	Da	1	0.306	1.342	0.23	0.64 (0.46, 0.82)	3.60E−05	0.89
E	B	1	0.673	2.282	0.3	0.83 (0.74, 0.91)	5.50E−15*	0.97
Ea	Eb	1	0.044	1.265	0.03	0.57 (0.42, 0.72)	3.64E−03	<0.05*
Eb	Ea	0.99	0.395	1.265	0.31	0.82 (0.73, 090)	3.64E−03	<0.05*
Ea3-4	Eb1-4	1	0.044	1.16	1	0.57 (0.42, 0.72)	3.64E−03	<0.05*
Eb1-4	Eb5-8	1	0.051	0.66	1	0.88 (0.76, 1.0)	1.98E−03	<0.05*
Eb5-8	Eb1-4	1	0.086	0.66	1	0.78 (0.64, 0.92)	1.98E−03	<0.05*
F +Ea1-2	B	0.99	0.113	2.319	0.05	0.92 (0.82, 1.00)	6.10E−13*	<0.05*
S	Da	1	0.254	2.52	0.1	0.96 (0.90, 1.00)	6.10E−06*	0.29
Sa	Sb	1	0.048	0.752	0.06	0.75 (0.57, 0.92)	1.10E−04	<0.05*
Sb	Sa	0.85	0.06	0.452	0.08	0.97 (0.91, 1.00)	1.10E−04	<0.05*
**B)**								
**MOTU**	**Closest Species**	***Clade Support***	***Intra***	***Inter***	***Intra/Inter***	***P ID(Strict)***	***Rosenberg's P_AB_***	***Rodrigo’s P(RD)***
A	B	89	0.012	0.206	0.06	0.97 (0.92, 1.00)	2.85E−03	0.06
C	B	100	0.003	0.264	0.01	0.98 (0.92, 1.00)	6.70E−08*	<0.05*
D	B	68	0.12	0.227	0.53	0.58 (0.45, 0.71)	5.60E−08*	1
Da	Db	98	0.001	0.187	0.01	0.79 (0.61, 0.96)	0.02	<0.05*
Db	Da	100	0.037	0.187	0.2	0.66 (0.48, 0.84)	0.02	0.35
E	C	100	0.095	0.319	0.3	0.82 (0.74, 0.91)	6.70E−08*	1
Ea	Eb	100	0.003	0.191	0.02	0.58 (0.43, 0.73)	3.64E−03	<0.05*
Eb	Ea	76	0.05	0.191	0.26	0.84 (0.76, 0.93)	3.64E−03	0.26
Ea3-4	Eb1-4	100	0.003	0.174	100	0.58 (0.43, 0.73)	3.64E−03	<0.05*
Eb1-4	Eb5-8	98	0.003	0.086	98	0.91 (0.79, 1.0)	1.98E−03	<0.05*
Eb5-8	Eb1-4	100	0.007	0.086	100	0.81 (0.67, 0.96)	1.98E−03	<0.05*
F +Ea1-2	B	98	0.009	0.313	0.03	0.93 (0.83, 1.00)	7.10E−12*	<0.05*
S	F +Ea1-2	100	0.028	0.539	0.05	0.98 (0.92, 1.00)	5.50E−21*	1
Sa	Sb	100	0.002	0.093	0.02	0.78 (0.60, 0.95)	1.10E−04	<0.05*
Sb	Sa	76	0.003	0.093	0.03	0.98 (0.93, 1.00)	1.10E−04	<0.05*

*Intra / Inter* – ratio of *Intra* (genetic differentiation among members of a putative species) to *Inter* (genetic differentiation between the members of a putative species and the members of the closest putative species), *P ID(Strict)* - mean (95% confidence interval) probability of correctly identifying an unknown member of a given clade using the criterion that it must fall within, but not sister to, the species clade in a tree, *Rosenberg’s P_AB_* - probability of reciprocal monophyly under a random coalescent model and *Rodrigo’s P(RD)* – probability that a clade has the observed degree of distinctiveness due to random coalescent processes.

* Significant values (values remained significant after Bonferroni correction).

**Table 8:** Illustrative selection of p- and K2P distance values for *Rumina* sp. and other Stylommatophora.

A)				
Gene	Species	p-distance	K2P distance	Reference
16S	*Rumina decollata* and *R. saharica*	0.165	0.188	present work
	*Albinaria* spp.		0.095	Douris et al, 1998 [103]
	*Arion fasciatus* and *A. silvaticus*	0.100		Geenenet al, 2006 [10]
	*A. fasciatus* and *A. circumscriptus*	0.036–0.097		Geenenet al, 2006 [10]
	*A. subfuscus* and *A. fuscus*	0.18–0.22		Pinceel et al, 2005 [104]
	*A. transsylvanus* and other *Arion* spp.		0.15–0.19	Jordaens et al, 2010 [105]
	*Leptaxis azorica* and *L. caldeirarum*	0.135		Van Riel et al, 2005 [106]
	*Mandarina anijimnan* and other		0.17	Chiba, 1999 [107]
	*Mandarina* spp.			
ITS1	*Rumina decollata* and *R. saharica*	0.023	0.023	present work
	*Arion subfuscus* and *A. fuscus*	0.013		Pinceel et al, 2005 [104]
	*Cochlicopa nitens, C. lubrica* and *C.*		0.005–0.023	Armbruster et al, 2000 [108]
	*lubricella*			
**B)**				
16S	*A. transsylvanus*		0.007	Jordaens et al, 2010 [105]
	*A. fuscus*		0.012	Jordaens et al, 2010 [105]
	*Candidula unifasciata*	0.049		Pfenninger and Posada,
				2002 [109]
	*Cepaea nemoralis*		0.13	Thomaz et al, 1996 [110]
	*Cepaea nemoralis*		0.06	Davison, 2000 [111]
	*Euhadra peliomphala*		0.10	Hayashi and Chiba, 2000
				[112]
	*Euhadra quaesita*		0.14	Watanabe and Chiba, 2001
				[113]
	*Helix aspersa*		0.13	Thomaz et al, 1996 [110]
	*Helix aspersa*	0.11		Guiller et al, 2001 [90]
	*Partula spp.*	0.10		Goodacre, 2002 [114]
COI	*Arianta arbustorum*	0.075		Haase et al., 2003 [115]
	*A. arbustorum*	0.18		Gittenberger et al., 2004
				[116]

Interspecific (A) and intraspecific (B) sequence divergences.

#### Genealogical sorting index

All MOTUs tested had a GSI value of 1.00 ( = monophyletic group) with both ML and BI, except for MOTUs D, E, Ea and F with BI (GSI = 0.82, 0.61, 0.27 and 0.70 respectively), and MOTUs E, Ea and F with ML (GSI = 0.57, 0.44, and 0.69 respectively). All values were statistically significant, even after sequential Bonferroni correction. Hence, GSI suggested eight to ten species with BI since both Eb and S were supported, either as single species, or as two species each (Eb1-4 and Eb5-8 for Eb and Sa and Sb for S), and seven to ten species with ML ( = BI result with MOTU D added as supported species (Table 6)).

#### General mixed yule coalescent model

The maximum likelihood of the null model (i.e. all sequences belong to a single species) was significantly lower than the maximum likelihood of the GMYC model, both when considering a single-speciation event [346 (for the null model) vs 367 (for the maximum likelihood of the GMYC), ratio: 40.7, p = 7.5×10^−9^], and multiple speciation events (346 vs 373, ratio: 53.2, p = 3.1×10^−10^). The threshold time at which the single speciation-coalescent transition occurred was 0.247 Mya, yielding ten putative species : A, C, Da, Db, Ea3-4, Eb1-4, Eb5-8, F+Ea1-2, Sa and Sb (Table 6). In case of the multiple speciation events, the threshold times at which the speciation-coalescent transitions occurred were 0.213, 0.072 and 0.011 Mya, yielding 17 putative species clusters.

## Discussion

### Species Delimitation Methods

We aimed at exploring the taxonomic status of the nominal *Rumina* species within a phylogenetic context in order to avoid the reproductive mode constraints imposed by the BSC. Yet, the barcoding gap analysis and ABGD are based on genetic distances only and do not use tree topologies. It is hence unclear to what extent these phenetic methods allow to delimit species. While this is particularly true for the ABGD method (for which the independence of a tree topology is seen as an advantage [50]), it seems less an issue with the barcode gap analysis since this approach can be applied as a post hoc evaluation of species level divergences among MOTU. In contrast, SDP, GSI and GMYC rely on the recognition of monophyletic groups and hence these methods should allow delimiting species under the PSC. Thus, by combining topology free (distance) methods with phylogeny dependent methods to delimit species, we assessed in how far these different approaches converge to an unequivocal, stable taxonomic interpretation. Yet, by suggesting a range of five to 17 putative species (Table 6) the different species delimitation methods appear to be inconsistent. As such, the putative species interpretation of several, well-supported clades may differ between the methods (Table 6). It is therefore relevant to briefly discuss these methods in more detail, before using them to interpret species delimitation in *Rumina* sp.

The reliability of DNA barcoding gap analysis depends on the separation of putative intra- and interspecific sequence divergences [1], [70], [71], i.e. the “DNA barcoding gap” [48], [49]. Various thresholds have been proposed to do so. For example, a sequence divergence of 3% has been suggested as boundary between intra- and interspecific COI differentiation [1]. Similarly, a threshold of 10× the mean intraspecific sequence divergence may point to a species level gap [49]. However, a general COI barcoding threshold is difficult to define for stylommatophoran snails, because intraspecific divergences in this group can be as high as 30%, while interspecific divergences can be as low as 1%, thus implying a high overlap between both [72]. Nevertheless, a threshold of 4% would minimize the number of false positive (intraspecific divergence misdiagnosed as heterospecific) and false negative (interspecific divergence misdiagnosed as conspecific) species identifications in stylommatophorans [72]. This premise relied on the observation that in stylommatophorans the mean intraspecific divergence (ca. 3%) is considerably lower than the mean interspecific divergence (ca. 12%) [72]. However, the 4% threshold still provoked a misidentification rate of 32% so that DNA barcoding gap analysis alone is unreliable for separating stylommatophoran species [72]. As a variant of the 10× rule, an interspecific COI threshold value of 3.2–4.1× the level of intraspecific variation has been proposed in Cypraeidae (cowries; Hypsogastropoda) [70]. Yet, whether this threshold is also suitable for other gastropods remains to be assessed. Anyway, not only the gap thresholds, but also the way in which the barcoding gap analysis is executed, may affect the outcome. For example, OGA vs PGA analysis of our data with a given threshold yielded seven to 10 putative species (Table 6).

It has been claimed that the ABGD method is more objective because it automatically finds the distance where a barcode gap is located [50]. So, it does not rely on a subjective choice of a threshold [50]. As such, in *Rumina* ABGD indicated Eb as a single species, whereas OGA and PGA further split Eb into the species Eb1-4 and Eb5-8 (Figure 4l and Figure 4m and Table 6).

The SDP method is expected to be a better predictor of species identity than DNA barcoding gap analyses, because the *Intra/Inter* ratio measures the amount of sequence divergence between a focal species and its closest relative [51]. The significant values of *Rosenberg’s P_AB_* for A, C, E, F+Ea1-2 and S in the BI tree and for C, D, E, F and S in the ML tree mean that the null hypothesis of “random coalescence” is rejected. Hence the monophyly of these MOTUs is not a chance result [60]. Yet, random coalescence could not be rejected for the other MOTUs in the BI and ML trees, because their *Rosenberg’s P_AB_* were not significant. Particularly for MOTUs A in the ML tree and Eb in the BI and ML trees, this result is surprising since these clades were strongly supported in the trees. Possibly, this result reflects the fact that *Rosenberg’s P_AB_* assumes panmixis [51], which may be not generally valid for *R. decollata* [25]. *Rodrigo’s P(RD)* also assumes panmixis [51] and values <0.05 indicate that panmixis does not hold [61], [73]. *Rodrigo’s P(RD)* were less conservative than *Rosenberg’s P_AB_* since MOTUs Da, Ea3-4, Eb1-4, Eb5-8, Sa and Sb were considered as putative species. Therefore, while *Rosenberg’s P_AB_* considered five putative species within *Rumina*, *Rodrigo’s P(RD)* considered eight to nine putative species. However, the statistical significance of *Rodrigo’s P(RD)* can be overestimated [73] since only one of many coalescent models is used in the calculations [61].

By suggesting seven to 10 putative species within *Rumina,* the GSI was less conservative than *Rosenberg’s P_AB_.* This is because the GSI can track divergence before monophyly is achieved, so that GSI is able to distinguish “young” species [52]. MOTUs Da, Db, Ea3-4, Eb1-4, Eb5-8, Sa and Sb may fit into this scenario.

Finally, the GMYC method suggested more putative species in *Rumina* (10 to 17 species) than the other methods. This tendency was noted before [74], [75], [76] and was attributed to incomplete sampling of demes involved in the coalescence process, so that artificial clusters are produced by which the number of putative species is overestimated [77]. It is hence not excluded that our incomplete sampling (particularly in North Africa) may have provoked such artificial taxonomic inflation.

Recapitulation of the species delimitation results in *Rumina* shows that only MOTUs C and F+Ea1-2 are retained as putative species by all methods. For the other MOTUs, the methods yielded variable interpretations (Table 6): (1) MOTU A was supported as putative species by all methods except the DNA barcoding gap analysis with the 10× rule and SDP (ML); (2) MOTUs D, Da and Db were inconsistently interpreted with either D (overall) as a single species (e.g. by *Rosenberg’s P_AB_* with the ML), only Da as a single species (e.g. barcoding gap analysis with the 3% and 10× thresholds), or Da and Db as two different species (e.g. ABGD, GSI, GMYC); (3) although MOTU E appeared as a single species by *Rosenberg’s P_AB_*, all other methods subdivided E in two (Ea 3-4 and Eb) or three (Ea 3-4, Eb1-4 and Eb5-8) species (Table 6); (4) *R. saharica* was suggested either as one species (S) (e.g. GSI and some barcode gap analyses) or as two species (Sa and Sb) (e.g. ABGD, GMYC, some barcode gap analyses). Such inconsistencies among species delimitation methods, resulting in different level of species distinctiveness have already been reported [73], [74], [78], [79]. So, to what extent are the species delimitation methods implemented here really helpful? If used to formulate species hypotheses, then the discrepancies between the methods are no problem, and perhaps even an advantage, because of the wider variety of hypotheses that can be produced by the different methods. Yet, all the species hypotheses suggested by the methods implemented here, involved a simple selection of well-supported clades in the trees. So, the added value of the species delimitation methods is not trivial. Moreover, given the discrepancies between the methods, it is difficult to decide which species delimitation method one must choose for analyzing a data set. It is not the place here to expand on this issue, but it must be clear that comparative evaluations of species delimitation methods are needed on both simulated data and well-known taxonomic groups, and in relation to well-defined species concepts. Indeed, the use of different species concepts may lead to conflicting taxonomic interpretations of the same groups of individuals [80].

We will now discuss the combined results of the species delimitation methods (COI) and the other phylogenetic data in order to propose a tentative taxonomic re-interpretation of *Rumina*.

### Rumina Saharica

*R. saharica* is conchologically and anatomically well-differentiated from *R. decollata* [22], [81], and has also a different geographic distribution (eastern part of the Mediterranean) [27]. The present DNA sequence data show that *R. saharica* is also a well-supported clade that is separated from *R. decollata* by mean sequence divergences that are of the same magnitude, or larger, than the corresponding divergences for other stylommatophoran species (Table 8). Hence, all species delimitation methods supported *R. saharica* as a separate species, an interpretation that was further corroborated by (1) the 21 cmtDNA and 15 cITS indels differentiating both taxa (Table 5), and (2) the well-supported reciprocal monophyly of *R. saharica* and *R. decollata* in the cmtDNA, cITS and species trees. Hence, *R. saharica* complies with the PSC and by extension the general lineage concept [19], [20]. Although some species delimitations methods further split *R. saharica* in two putative species (Sa and Sb), these MOTUs showed no additional diagnostic differences. Therefore, we currently do not consider them as different species, but interpret them as intraspecific (geographic) differentiation.

### Rumina Paivae

The DNA sequence data do not support *R. paivae* as a species under the PSC and the lineage concept, since the *R. paivae* haplotypes did not form a clade, but were distributed over three clades within *R. decollata.* The two shells used to describe *Bulimus paivae* were 39–44 mm long and 16 mm wide, while much later it was specified that the body-whorl of *R. paivae* has a width of 15.5–22.7 mm [29]. Thus *R. paivae* is mainly defined by its large size compared to *R. decollata* (shell width: 6.5–16.5 mm; shell length: 18–40 mm [29], [82]). Hence, we currently regard *R. paivae* as a large phenotype within the size range of several MOTUs of *R. decollata*. As such it contrasts with another North African “giant” shell phenotype, viz. *Cornu aspersum maxima* (Taylor, 1883). This latter taxon was originally described as a large form of *Cornu aspersum* (Müller, 1774), but was subsequently raised to subspecific rank because of its well-defined allopatric distribution (Morocco), its genital morphology [83], [84], shell morphometrics [85], allozyme differentiation ([86], [87], [88] but see [89]), and 16S sequence divergence [90]. The 16S data even suggested that *C. a. maxima* forms a well-defined sister clade to *C. a. aspersum* and thus may even represent a diagnosable species under a PSC (even if, as far as we know, no one has hitherto gone so far as to raise *C. a. maxima* to a full species rank). Yet, *R. paivae* does not show this sort of consistent taxonomic differentiation with respect to *R. decollata*.

### Rumina Decollata

**Figure 5:**
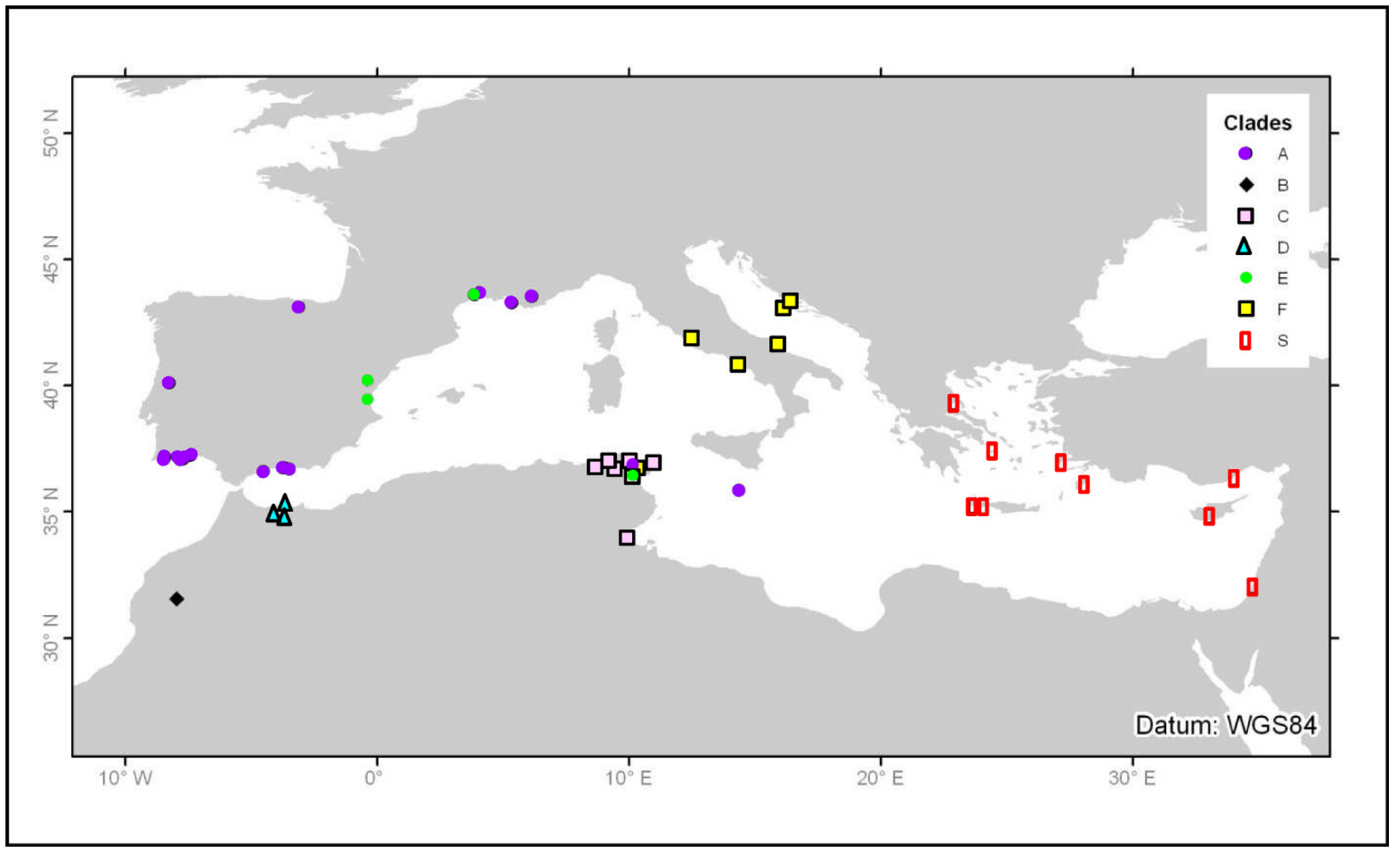
Geographic distribution of *Rumina* haplotypes.

There is a strong phylogenetic structuring within *R. decollata*, since the cmtDNA data show that this taxon comprises several well-supported MOTUs, some of which are almost as strongly divergent as *R. decollata* and *R. saharica* (Table 4). For MOTUs A and Eb this phylogenetic structuring is corroborated by: (1) clade-specific differences at 12 cmtDNA and 4 ITS indels (Table 5), (2) the monophyly of MOTU A with cITS (MOTU Eb is not supported by cITS data), (3) all the species delimitation methods for MOTU A (except for SDP with ML) and by a majority of species delimitation methods for MOTU Eb, and (4) by a consistent body color difference, with MOTU A being dark and MOTU Eb being light [16]. Because both MOTUs occur sympatrically, their DNA and color differences do not reflect current geographic structuring, but rather point to an evolutionary split suggesting that both MOTUs are different species under a PSC. Obviously, if MOTUs A and Eb are regarded as valid species, then there is no a priori reason to deny the other *R. decollata* clades a species level interpretation as well. Even the single MOTU B, which is separated from the other MOTUs by eight rDNA indels and which forms a well-supported clade in cITS, can tentatively be regarded as a species. However, currently these MOTUs are only diagnosed on the basis of the present DNA sequence data. Therefore our suggestion to regard them as species under a PSC needs further exploration. This also follows from the observation that several MOTUs show more or less complementary distributions suggesting substantial geographic structuring. Indeed, roughly four MOTU areas can be recognized, viz. the Iberian Peninsula and Southern France (MOTUs A and Eb), Italy and Western Balkan (MOTU F), Western Morocco (B), Northeastern Morocco (D) and Tunisia (C, Ea with one haplotype from MOTU F and one from MOTU A) (Figure 5). Thus, despite our still limited geographic sampling and the risk that this may have induced artificial phylogenetic structuring [77], it seems as if *R. decollata* shows a similar sort of complex phylogeographic pattern as several other Mediterranean land snail taxa such as *Cornu aspersum* [90], [91], *Tudorella* [92] and *Sphincterochila* [93], in which deep phylogeographic subdivisions have been associated with (cryptic) taxonomic differentiation. Hence, further intensive sampling of *R. decollata*, particularly in North Africa, is needed to further explore the phylogeographic subdivision and taxonomy of this species complex.

It will be interesting to combine other types of data (e.g. morphological, ecological, life-history, …) under an ‘integrative taxonomical framework’ (e.g. [74], [94], [95], [96]) in *Rumina*.

### Conclusion

The present phylogenetic interpretation of *Rumina* sp., based on mtDNA and ITS sequence data, supports the species level status of *R. saharica* and suggests that at least six MOTUs in *R. decollata* are putative species. Yet, we found no support for *R. paivae*. Hence, we tentatively suggest that *Rumina* is a complex of at least seven phylogenetic species: *R. saharica*, the dark (A) and light (Eb) color phenotypes of *R. decollata*, three North African species (B, C and D), and an Italian-Croatian species (F). These putative species need further corroboration by an integrative taxonomic approach, combined with a more comprehensive geographic sampling. This should allow to (1) diagnose the species and formalize their nomenclature, (2) verify their status under other species concepts, and (3) reconstruct their biogeographic history. From a methodological point of view, the present paper illustrates that species delimitation methods applied to COI DNA barcodes may be inconsistent as they produce alternative species hypotheses for the same data. It is hence difficult to choose a priori which method to implement, and since each method only yields a subset of all species hypotheses implied by a phylogenetic tree, one may wonder whether species delimitation methods have an added value and whether it is not simpler to propose (phylogenetic) species hypotheses by referring to well-supported clades.

## Supporting Information

Figure S1 **BI tree of** ***Rumina*** **based on the COI sequences.** BI posterior probabilities are shown near the nodes. Haplotypes are listed in Table S1.
(TIF)

Figure S2 **ML tree of** ***Rumina*** **based on the COI sequences.** ML bootstrap values are shown near the nodes. Haplotypes are listed in Table S1.
(TIF)

Table S1 **Geographic origins of the material studied.**
(DOCX)
