# Supplementary figures and images for: Exploring Species Level Taxonomy and Species Delimitation Methods in the Facultatively Self-Fertilizing Land Snail Genus Rumina (Gastropoda: Pulmonata)
Source: PLoS One. 2013 Apr 5;8(4):e60736. doi: 10.1371/journal.pone.0060736 (PMC3618274; doi:10.1371/journal.pone.0060736)

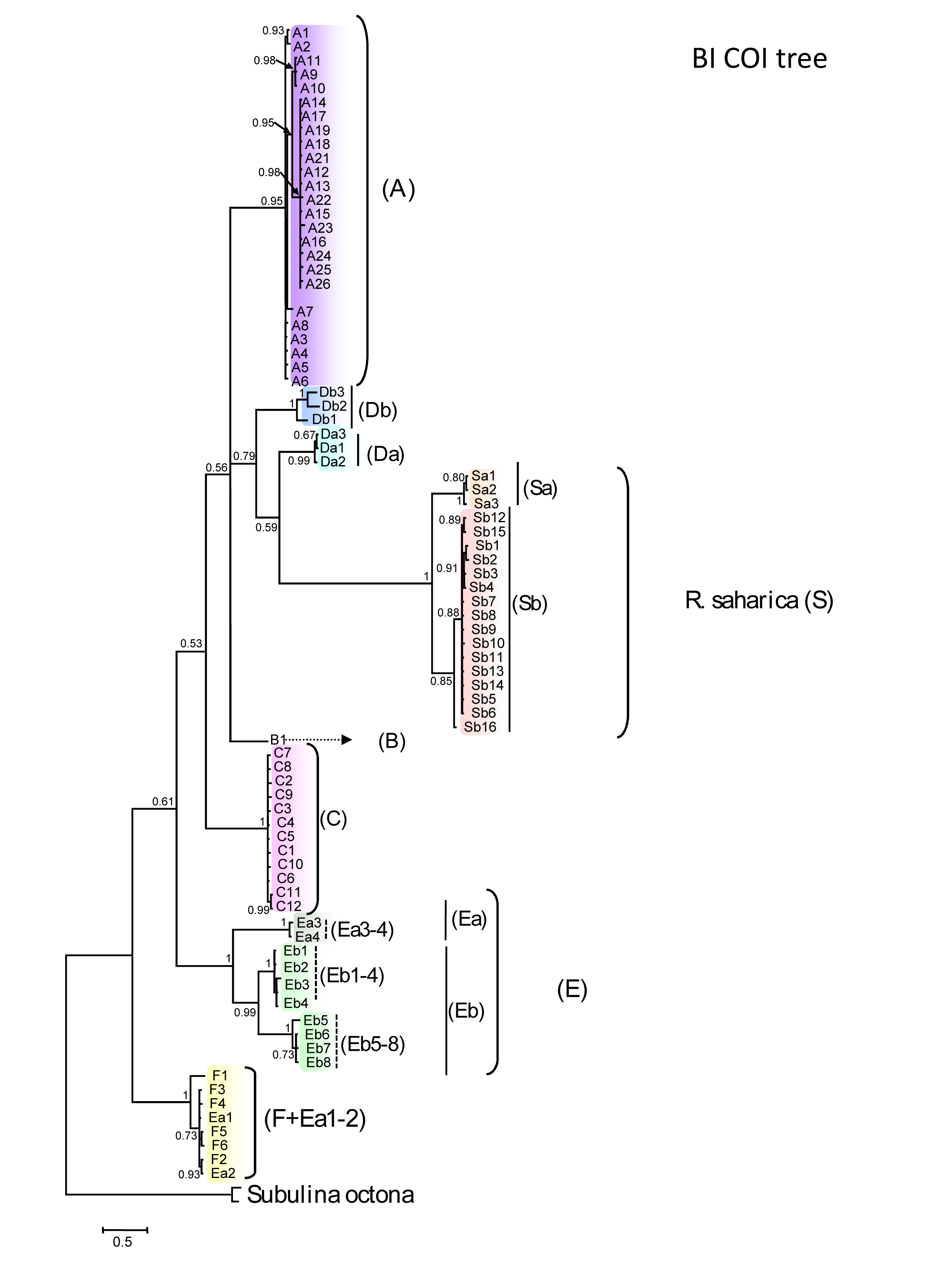

Supplement: Figure S1 — BI tree of Rumina based on the COI sequences. BI posterior probabilities are shown near the nodes. Haplotypes are listed in Table S1. (TIF) [file pone.0060736.s001.tif]

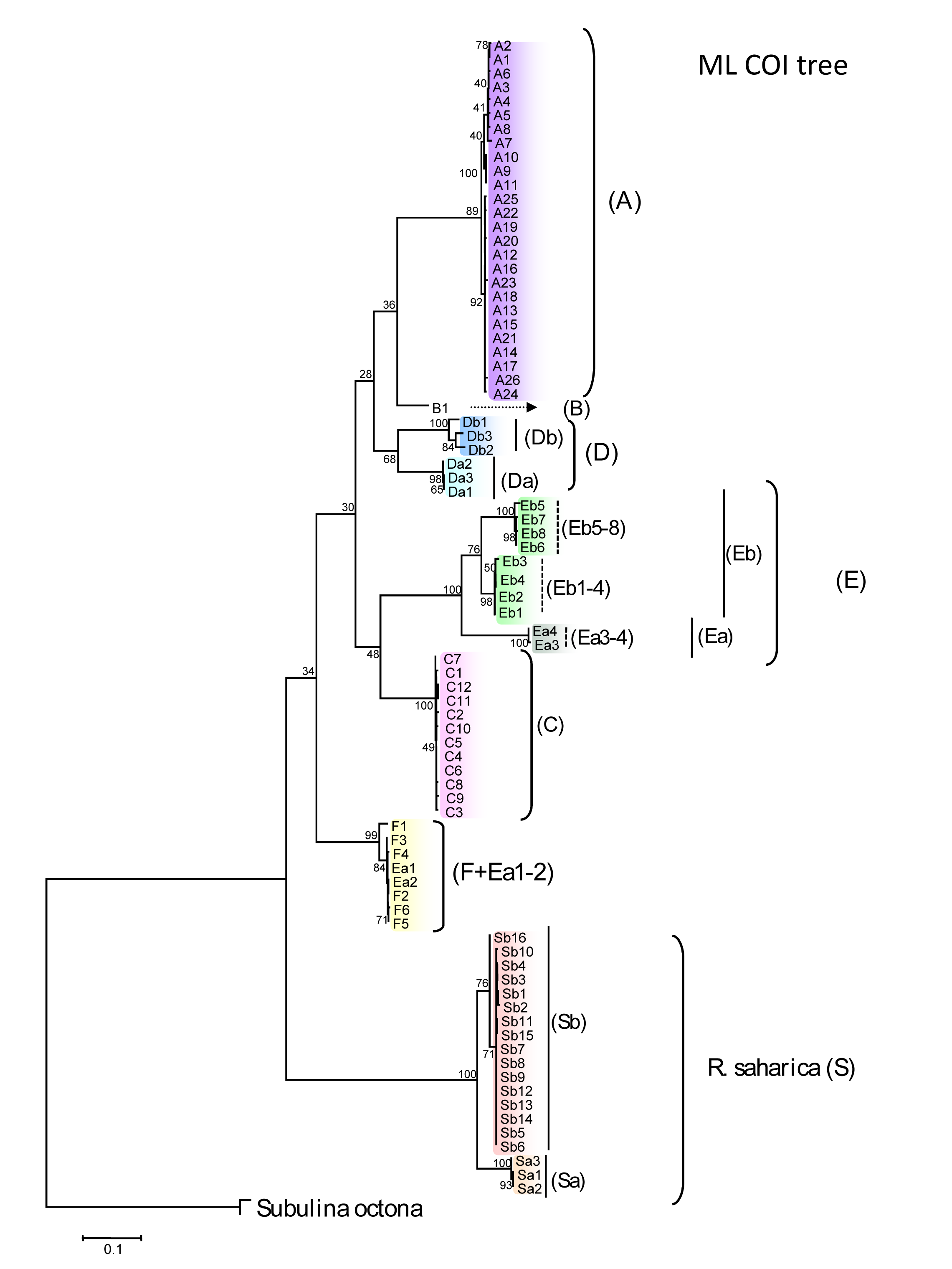

Supplement: Figure S2 — ML tree of Rumina based on the COI sequences. ML bootstrap values are shown near the nodes. Haplotypes are listed in Table S1. (TIF) [file pone.0060736.s002.tif]
