# Supplementary material for: Exploring Species Level Taxonomy and Species Delimitation Methods in the Facultatively Self-Fertilizing Land Snail Genus Rumina (Gastropoda: Pulmonata)
Source: PLoS One. 2013 Apr 5;8(4):e60736. doi: 10.1371/journal.pone.0060736 (PMC3618274; doi:10.1371/journal.pone.0060736)
Supplement: Table S1 — Geographic origins of the material studied. (DOCX) [file pone.0060736.s003.docx]

**Table S1.** Geographic origins of the material studied.

| **Country** | **Locality** | **Name** | **N** | **H_cmtDNA_** | **H_cITS_** | **Sp** | **GenBank accession numbers** | | | | | | **Leg.** |
| --- | --- | --- | --- | --- | --- | --- | --- | --- | --- | --- | --- | --- | --- |
|  |  |  |  |  |  |  | **12S** | **16S** | **COI** | **CytB** | **ITS1** | **ITS2** |  |
| Algeria | Unknown | Alg1 | 8 | C6 | ns | D | JX988237 | JX988321 | JX988034 | JX988118 | ns | ns | Noureddine Soltani, |
|  | Unknown | Alg2 | 8 | Ea4 | nEa3 | D | JX988253 | JX988337 | JX988050 | JX988134 | JX988177 | JX988380 | Guy Smagghe |
| Croatia | Komiza, Vis Island | Cro2 | 11 | F3 | nF1 | D | JX988264 | JX988348 | JX988061 | JX988145 | JX988179 | JX988382 | Raoul Van Damme |
|  | Milna, Brac Island | Cro1 | 4 | F5 | nF5 | D | JX988266 | JX988350 | JX988063 | JX988147 | JX988183 | JX988386 | Vesna Stamol |
| Cyprus | Lara | Cypr1 | 1 | Sb16 | nS1 | D | JX988204 | JX988288 | JX988001 | JX988085 | JX988151 | JX988354 | Katerina Vardinoyannis |
| France | Cotignac | FM1 | 11 | A10 | nA2 | D | JX988214 | JX988298 | JX988011 | JX988095 | JX988155 | JX988358 | Vanya Prévot, Renaud Hecq, Marion de Bast, Gontran Sonet |
|  | Marseille | FM2 | 5 | A22 | nA2(1),nA6(2), | D | JX988226 | JX988310 | JX988023 | JX988107 | JX988155, | JX988358, | Janine and Jean |
|  |  |  |  |  | nA7(2) |  |  |  |  |  | JX988159- | JX988362- | Demartini |
|  |  |  |  |  |  |  |  |  |  |  | 60 | 3 |  |
|  | Marseille | FM3 | 10 | A9(7), | nA2(7), | D | JX988213, | JX988297, | JX988010, | JX988094, | JX988155, | JX988358, |  |
|  |  |  |  | Eb5(3) | nEb1(3) |  | JX988258 | JX988342 | JX988055 | JX988139 | JX988171 | JX988374 |  |
|  | Montpellier Arceaux 1 | FmtA1 | 10 | Eb1(7),Eb2(3) | nEb2 | D | JX988254-5 | JX988338-9 | JX988051-2 | JX988135-6 | JX988172 | JX988375 | Vanya Prévot, Gontran Sonet |
|  | Montpellier Arceaux 2 | FmtA2 | 10 | Eb1 | nEb2 | D | JX988254 | JX988339 | JX988051 | JX988135 | JX988172 | JX988375 |  |
|  | Montpellier | FmtB1 | 13 | A11(8), Eb1(1), | hetero | D | JX988215, | JX988299, | JX988012, | JX988096, |  |  |  |
|  | Botanique 1 |  |  |  |  |  |  |  | JX988051, | JX988135, |  |  |  |
|  |  |  |  |  |  |  |  |  | JX988052 | JX988136 |  |  |  |
|  |  |  |  | Eb2(4) |  |  | JX988254, | JX988338, |  |  |  |  |  |
|  |  |  |  |  |  |  | JX988255 | JX988339 |  |  |  |  |  |
|  | Montpellier | FmtB2 | 26 | A11(25),Eb1(1) | nA2(1), | D | JX988215, | JX988299, | JX988012, | JX988096, | JX988155, | JX988358, |  |
|  | Botanique 2 |  |  |  | nEb2(25) |  | JX988254 | JX988338 | JX988051 | JX988135 | JX988172 | JX988375 |  |
|  | Montpellier | FmtC | 20 | A11(2),Eb1(16), | nA2(2), | D | JX988215, | JX988299, | JX988012, | JX988096, | JX988155, | JX988358, |  |
|  |  |  |  |  |  |  |  | JX988338, | JX988051, | JX988135, | JX988172 | JX988375 |  |
|  |  |  |  |  |  |  |  | JX988341 | JX988054 | JX988138 |  |  |  |
|  | Cimetière 1 |  |  | Eb4(2) | nEb2(18) |  | JX988254, |  |  |  |  |  |  |
|  |  |  |  |  |  |  | JX988257 |  |  |  |  |  |  |
|  | Montpellier Lycée de Joffre | FmtL | 13 | Eb4 | nEb2 | D | JX988257 | JX988341 | JX988054 | JX988138 | JX988172 | JX988375 |  |
|  | Montpellier Spot 1 | FmtS | 12 | Eb1 | hetero | D | JX988254 | JX988338 | JX988051 | JX988135 |  |  |  |
| Greece | Kithnos Island | Grc3 | 2 | Sa2 | nS1 | S | JX988187 | JX988271 | JX987983 | JX988068 | JX988151 | JX988354 | Constantine Mifsud, Panayotis Ovalis |
|  | Lindos (Rhodos) | Grc1 | 6 | Sb1(2),Sb2(1), | nS1 | S | JX988189- | JX988273- | JX987986- | JX988070- | JX988151 | JX988354 |  |
|  |  |  |  | Sb3(1),Sb4(1), |  |  | 92, | 6, | 9, | 3, |  |  |  |
|  |  |  |  | Sb14(1) |  |  | JX988202 | X988286 | JX987999 | JX988083 |  |  |  |
|  | Lindos (Rhodos) | Grc7 | 3 | Sb10(1),Sb11(1), | nS3 | S | JX988198- | JX988282- | JX987995- | JX988079- | JX988153 | JX988356 |  |
|  |  |  |  | Sb12(1) |  |  | 200 | 4 | 7 | 81 |  |  |  |
|  | Lindos (Rhodos) | Grc8 | 3 | Sb7(1),Sb8(1), | nS3 | S | JX988195- | JX988279- | JX987992- | JX988076- | JX988153 | JX988356 |  |
|  |  |  |  | Sb9(1) |  |  | 7 | 81 | 4 | 78 |  |  |  |
|  | Selinou | Grc5 | 9 | Sb16 | nS1 | S | JX988204 | JX988288 | JX988001 | JX988085 | JX988151 | JX988354 |  |
|  | Siteias, Cape Sidero | Grc4 | 3 | Sb15 | nS1 | S | JX988203 | JX988287 | JX988000 | JX988084 | JX988151 | JX988354 |  |
|  | Volou Alykes | Grc6 | 6 | Sa1(1),Sa2(1), | nS1 | S | JX988186- | JX988270- | JX987983- | JX988067- | JX988151 | JX988354 |  |
|  |  |  |  | Sa3(4) |  |  | 8 | 2 | 5 | 9 |  |  |  |
| Israel | Tel Aviv | Isr1 | 10 | Sb13 | nS2 | S | JX988201 | JX988285 | JX987998 | JX988082 | JX988152 | JX988355 | Uri Bar-Zeev, Henk K. Mienis |
| Italy | Gargano | ItG | 7 | F1 | nF2 | D | JX988262 | JX988346 | JX988059 | JX988143 | JX988180 | JX988383 | Kurt Jordaens |
|  | Napoli | ItNp1 | 11 | F4 | nF3 | D | JX988265 | JX988349 | JX988062 | JX988146 | JX988181 | JX988384 | Paolo Alfonso Pedata |
|  | Roma | ItRo1 | 2 | F6 | nF6 | D | JX988267 | JX988351 | JX988064 | JX988148 | JX988184 | JX988387 | Vanya Prévot, Gontran Sonet |
| Malta | Dingli | Mt1 | 19 | A23 | nA9 | D | JX988227 | JX988311 | JX988024 | JX988108 | JX988162 | JX988365 | Constantine Mifsud |
| Morocco | Aazanèn | Mrc3 | 2 | Db2(1),Db3(1) | nD2(1),nD3(1) | P | JX988248- | JX988332- | JX988045- | JX988129- | JX988169- | JX988371- | Mohamed Ghamizi |
|  |  |  |  |  |  |  | 9 | 3 | 6 | 30 | 70 | 3 |  |
|  | Beni Snassen | Mrc1 | 3 | Da1(1),Da2(1), | nD1 | D | JX988244- | JX988328- | JX988041- | JX988125- | JX988168 | JX988371 | Cédric Audibert, |
|  |  |  |  | Da3(1) |  |  | 6 | 30 | 3 | 7 |  |  | Harold Labrique |
|  | Marrakech | Mrc4 | 14 | B1 | nB1(9),nB2(5) | D | JX988231 | JX988315 | JX988028 | JX988112 | JX988163, | JX988366, | Mohamed Ghamizi |
|  |  |  |  |  |  |  |  |  |  |  | JX988164 | JX988367 |  |
|  | Tafersite | Mrc2 | 1 | Db1 | nD2 | D | JX988247 | JX988331 | JX988044 | JX988128 | JX988169 | JX988372 |  |
| Portugal | Carvoeiro | PCa1 | 7 | A13 | ns | D | JX988217 | JX988301 | JX988014 | JX988098 | ns | ns | Vanya Prévot, Zoltan Nagy, Gontran Sonet |
|  | Carvoeiro | PCa2 | 8 | A13 | ns | D | JX988217 | JX988301 | JX988014 | JX988098 | ns | ns | Charlotte Havermans, Gontran Sonet, Vanya Prévot |
|  | Laranjeiro | PL2 | 5 | A5 | nA3(2),nA4(3) | D | JX988209 | JX988293 | JX988006 | JX988090 | JX988156- | JX988359- | Vanya Prévot |
|  |  |  |  |  |  |  |  |  |  |  | 7 | 60 |  |
|  | Laranjeiro | PL3 | 3 | A5 | ns | D | JX988209 | JX988293 | JX988006 | JX988090 | ns | ns |  |
|  | Laranjeiro | PL4 | 2 | A5 | ns | D | JX988209 | JX988293 | JX988006 | JX988090 | ns | ns |  |
|  | Lousã | PLo | 8 | A8 | ns | D | JX988212 | JX988296 | JX988009 | JX988093 | ns | ns | Paula Cristina Ramalheiro Lourenço |
|  | Luz de Tavira | PLt1 | 3 | A3 | nA4 | D | JX988207 | JX988291 | JX988004 | JX988088 | JX988157 | JX988360 | Cláudia Patrão |
|  | Luz de Tavira | PLt2 | 2 | A4 | nA4 | D | JX988208 | JX988292 | JX988005 | JX988089 | JX988157 | JX988360 |  |
|  | Moncarapacho | PM1 | 2 | A5(1),A12(1) | nA3(1),nA4(1) | D | JX988209, | JX988293, | JX988006, | JX988090, | JX988156- | JX988359- | Vanya Prévot, Jean-Pierre |
|  |  |  |  |  |  |  | JX988216 | JX988300 | JX988013 | JX988097 | 7 | 60 | Prévot, Monique Lesenfants Prévot |
|  | Moncarapacho | PM2 | 10 | A13 | nA3 | D | JX988217 | JX988301 | JX988014 | JX988098 | JX988156 | JX988359 | Sarah Prévot |
|  | São Brás de Alportel | PSB | 7 | A13 | nA3 | D | JX988217 | JX988301 | JX988014 | JX988098 | JX988156 | JX988359 | Hélia Jacinto |
|  | Silves | PSi | 8 | A1(1),A2(1), | nA4 | D | JX988205-, | JX988289- | JX988002- | JX988086- | JX988157 | JX988360 | Charlotte Havermans, |
|  |  |  |  | A7(2),A26(4) |  |  | 6 | 90, | 3, | 7, |  |  | Gontran Sonet, |
|  |  |  |  |  |  |  | JX988211, | JX988295, | JX988008, | JX988092, |  |  | Vanya Prévot |
|  |  |  |  |  |  |  | JX988230 | JX988314 | JX988027 | JX988111 |  |  |  |
|  | Tavira | PTv | 11 | A4(5),A21(6) | nA3(6),nA4(5) | D | JX988208, | JX988292, | JX988005, | JX988089, | JX988156, | JX988359- | Cláudia Patrão |
|  |  |  |  |  |  |  | JX988225 | JX988309 | JX988022 | JX988106 | JX988157 | 60 |  |
| Spain | Almuñécar | SAl1 | 2 | A15(1),A19(1) | nA8 | D | JX988219, | JX988303, | JX988016, | JX988100, | JX988161 | JX988364 | Alex Vanhaelen |
|  |  |  |  |  |  |  | JX988223 | JX988307 | JX988020 | JX988104 |  |  |  |
|  | Almuñécar | SAl2 | 4 | A17 | nA8 | D | JX988221 | JX988305 | JX988020 | JX988102 | JX988161 | JX988364 |  |
|  | Artziniega | Sar | 16 | A14 | nA3 | D | JX988218 | JX988302 | JX988015 | JX988099 | JX988156 | JX988359 | Arantzazu Elejalde |
|  | Ayamonte | SA | 3 | A6(2),A12(1) | nA1 | D | JX988210, | JX988294, | JX988007, | JX988091, | JX988154 | JX988357 | Vanya Prévot, Jean-Pierre |
|  |  |  |  |  |  |  | JX988216 | JX988300 | JX988013 | JX988094 |  |  | Prévot, Monique Lesenfants Prévot |
|  | Benalmadena | SBe | 9 | A20(4),A25(5) | nA8 | D | JX988224, | JX988308, | JX988021, | JX988105, | JX988161 | JX988364 | Serge Gofas |
|  | (Malaga) |  |  |  |  |  | JX988229 | JX988313 | JX988026 | JX988110 |  |  |  |
|  | La Herradure | SHe | 3 | A16 | ns | D | JX988220 | JX988304 | JX988017 | JX988101 | ns | ns | Alex Vanhaelen |
|  | Los Mases, | SC2 | 5 | Eb8 | nEb3(1), | D | JX988261 | JX988345 | JX988058 | JX988142 | JX988173- | JX988376- | Sergio Montagud Alario |
|  | Castellón |  |  |  | nEb4(4) |  |  |  |  |  | 4 | 7 |  |
|  | Mas de Borràs, | SC1 | 6 | Eb6(3),Eb7(3) | nEb3 | D | JX988259- | JX988343- | JX988056- | JX988140- | JX988173 | JX988376 | Sergio Montagud Alario |
|  | Castellón |  |  |  |  |  | 60 | 4 | 7 | 1 |  |  |  |
|  | Torrenueva de | STo | 3 | A18 | ns | D | JX988222 | JX988306 | JX988019 | JX988103 | ns | ns | Alex Vanhaelen |
|  | Motril |  |  |  |  |  |  |  |  |  |  |  |  |
|  | Valencia | SV2 | 8 | Eb3 | nEb2 | D | JX988256 | JX988340 | JX988053 | JX988137 | JX988172 | JX988375 |  |
| Tunisia | Béja | Tun6 | 4 | C8 | nC1 | D | JX988239 | JX988323 | JX988036 | JX988120 | JX988165 | JX988368 | Anonimus |
|  | Beni Metir | Tun9 | 6 | C4(1),C5(5) | nC3 | D | JX988235- | JX988319- | JX988032- | JX988116- | JX988167 | JX988370 |  |
|  |  |  |  |  |  |  | 6 | 20 | 3 | 7 |  |  |  |
|  | Bir El Bey | Tun5 | 2 | Ea2(1),F2(1) | nEa4(1),ns | DP | JX988250, | JX988335, | JX988048, | JX988132, | JX988178 | JX988381 |  |
|  |  |  |  |  |  |  | JX988263 | JX988347 | JX988060 | JX988144 |  |  |  |
|  | Dar chichou | Tun8 | 1 | C3 | nC3 | D | JX988234 | JX988318 | JX988031 | JX988115 | JX988167 | JX988370 |  |
|  | Oudhna | Tun7 | 2 | C2(1),C9(1) | nC2 | D P | JX988233, | JX988317, | JX988030, | JX988114, | JX988166 | JX988369 |  |
|  |  |  |  |  |  |  | JX988240 | JX988324 | JX988037 | JX988121 |  |  |  |
|  | Oued sejnene | Tun10 | 3 | C1(1),C10(1), | nC2 | D | JX988232, | JX988316, | JX988029, | JX988113, | JX988166 | JX988369 |  |
|  |  |  |  | C12(1) |  |  | JX988241, | JX988325, | JX988038, | JX988122, |  |  |  |
|  |  |  |  |  |  |  | JX988243 | JX988327 | JX988040 | JX988124 |  |  |  |
|  | Oasis Tozeu | Tun12 | 6 | C8 | nC2 | D | JX988239 | JX988323 | JX988036 | JX988120 | JX988166 | JX988369 |  |
|  | Tunis | Tun4 | 7 | A24 | nA5 | D | JX988228 | JX988312 | JX988025 | JX988109 | JX988158 | JX988361 |  |
|  | Utique | Tun11 | 3 | C1(2),C11(1) | nC2(2),nC3(1) | D | JX988232, | JX988316, | JX988029, | JX988113, | JX988166- | JX988369- |  |
|  |  |  |  |  |  |  | JX988242 | JX988326 | JX988039 | JX988123 | 7 | 70 |  |
|  | Zaghouan | Tun1 | 5 | C7(1),Ea3(4) | nC2(1), | D | JX988238, | JX988322, | JX988035, | JX988119, | JX988166, | X988369, |  |
|  |  |  |  |  | nEa2(4) |  | JX988252 | JX988336 | JX988049 | JX988133 | JX988176 | JX988379 |  |
|  | Zaghouan2 | Tun2 | 2 | Ea3 | nEa2 | D | JX988252 | JX988336 | JX988049 | JX988133 | JX988176 | JX988379 |  |
|  | Zaghouan3 | Tun3 | 3 | Ea1(1),Ea3(2) | nEa1(2),nF4(1) | D | JX988250, | JX988334, | JX988047, | JX988131, | JX988175, | JX988378, |  |
|  |  |  |  |  |  |  |  |  |  | JX988133 | JX988182 | JX988385 |  |
|  |  |  |  |  |  |  | JX988252 | JX988336 | JX988049 |  |  |  |  |
| Turkey | Mersin, Tasucu | Tt1 | 16 | Sb5(15),Sb6(1) | nS3 | S | JX988193- | JX988277- | JX987990- | JX988074- | JX988153 | JX988356 | Ümit Kebapçi, |
|  |  |  |  |  |  |  | 4 | 8 | 1 | 5 |  |  | Zeki Yildirim |
|  | **Total** | 68 | 458 |  |  |  |  |  |  |  |  |  |  |

In parentheses the numbers of specimens per haplotype

N - number of specimens sequenced

H_cmtDNA_ - concatenated mtDNA haplotype

H_cITS_ - concatenated ITS haplotypes

ns - not sequenced

Sp - species

D - *R. decollata*

P - *R. paivae*

S - *R. saharica*
